# Integrated, Transparent Silicon Carbide Electronics and Sensors for Radio Frequency Biomedical Therapy

**DOI:** 10.1021/acsnano.2c03188

**Published:** 2022-07-11

**Authors:** Tuan-Khoa Nguyen, Sharda Yadav, Thanh-An Truong, Mengdi Han, Matthew Barton, Michael Leitch, Pablo Guzman, Toan Dinh, Aditya Ashok, Hieu Vu, Van Dau, Daniel Haasmann, Lin Chen, Yoonseok Park, Thanh Nho Do, Yusuke Yamauchi, John A. Rogers, Nam-Trung Nguyen, Hoang-Phuong Phan

**Affiliations:** † Queensland Micro and Nanotechnology Centre, Griffith University, Brisbane, Queensland 4111, Australia; ‡ Department of Biomedical Engineering, College of Future Technology, Peking University, Beijing 100871, China; § School of Nursing and Midwifery, Griffith University, Brisbane, Queensland 4111, Australia; ∥ Menzies Health Institute Queensland, Brisbane, Queensland 4222, Australia; ⊥ Centre for Future Materials, University of Southern Queensland, Toowoomba, Queensland 4305, Australia; # Australian Institute for Bioengineering and Nanotechnology, The University of Queensland, Brisbane, Queensland 4072, Australia; ∇ School of Engineering and Built Environment, Griffith University, Brisbane, Queensland 4215, Australia; ○ State Key Laboratory for Mechanical Behavior of Materials, School of Materials Science and Engineering, Xi’an Jiaotong University, Xi’an 710049, Shaanxi, People’s Republic of China; ⧫ Querrey Simpson Institute for Bioelectronics, Northwestern University, Evanston, Illinois 60208, United States; ¶ Department of Advanced Materials Engineering for Information and Electronics, Kyung Hee University, Yongin 17104, Republic of Korea; + Graduate School of Biomedical Engineering, The University of New South Wales, Sydney, New South Wales 2032, Australia; ■ JST-ERATO Yamauchi Materials Space-Tectonics Project, Kagami Memorial Research Institute for Science and Technology, Waseda University, Tokyo 169-0051, Japan; % Department of Materials Science and Engineering, Department of Mechanical Engineering, Department of Biomedical Engineering, Departments of Electrical and Computer Engineering and Chemistry, and Department of Neurological Surgery, Northwestern University, Evanston, Illinois 60208, United States; ▲ School of Mechanical and Manufacturing Engineering, The University of New South Wales, Sydney, New South Wales 2052, Australia

**Keywords:** Bio-Integrated Electronics, Functional Endoscopy, Irreversible Electroporation, Radio Frequency Ablation, Silicon Carbide, Thermal Ablation

## Abstract

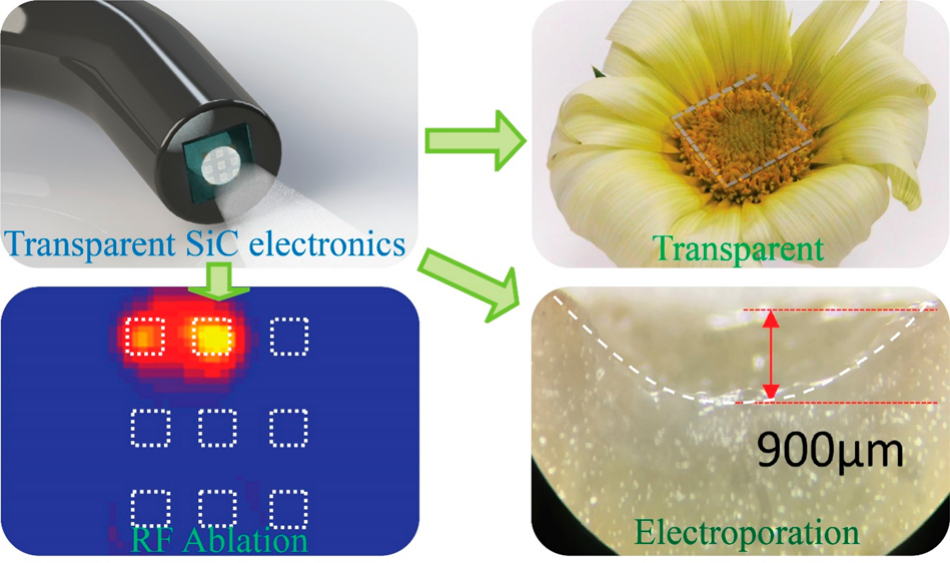

The integration of micro- and nanoelectronics into or onto biomedical devices can facilitate advanced diagnostics and treatments of digestive disorders, cardiovascular diseases, and cancers. Recent developments in gastrointestinal endoscopy and balloon catheter technologies introduce promising paths for minimally invasive surgeries to treat these diseases. However, current therapeutic endoscopy systems fail to meet requirements in multifunctionality, biocompatibility, and safety, particularly when integrated with bioelectronic devices. Here, we report materials, device designs, and assembly schemes for transparent and stable cubic silicon carbide (3C-SiC)-based bioelectronic systems that facilitate tissue ablation, with the capability for integration onto the tips of endoscopes. The excellent optical transparency of SiC-on-glass (SoG) allows for direct observation of areas of interest, with superior electronic functionalities that enable multiple biological sensing and stimulation capabilities to assist in electrical-based ablation procedures. Experimental studies on phantom, vegetable, and animal tissues demonstrated relatively short treatment times and low electric field required for effective lesion removal using our SoG bioelectronic system. *In vivo* experiments on an animal model were conducted to explore the versatility of SoG electrodes for peripheral nerve stimulation, showing an exciting possibility for the therapy of neural disorders through electrical excitation. The multifunctional features of SoG integrated devices indicate their high potential for minimally invasive, cost-effective, and outcome-enhanced surgical tools, across a wide range of biomedical applications.

Tissue ablation techniques employ locally applied energy to remove targeted tumors, through mechanisms that range from irreversible cell injury to tumor apoptosis.^1^ Thermal ablation and irreversible electroporation (IRE) are widely employed as powerful tools for minimally invasive tumor removal procedures, based in part on advances in integrated bioelectronics. Radiofrequency ablation (RFA) is one of the most effective thermal ablation techniques for removing cancerous tumors (e.g., in lungs, breasts, and kidneys), addressing tissue dysfunction, and treating cardiac disorders.^2^ Conventional RFA and IRE procedures typically incorporate a tip, often with expandable needles, and a collection of grounding pads that adhere to the skin to form a bipolar electrical configuration. These methods can enable percutaneous ablations with anatomical targets of tissue and/or tumor lesions.

To deliver the tissue ablation in the radio frequency range (e.g., 400–600 kHz), the required voltage and current can reach up to 60 V and 1 A (peak-to-peak values), respectively. This voltage/current range has been proven in maintaining safe operations in animal and human studies.^3,4^ The high frequency range and generated RF power aim to increase the tissue temperature while minimizing muscle contraction and pain to patients.^5^ For electroporation, the common applied peak-to-peak voltage is up to 200 V at a relatively low frequency of below 100 Hz. As only short voltage pulses are used in IRE (e.g., 100 μs pulse at 1 to 10 Hz), the power consumption and the side effects will be minimized.^6^ Even though several RFA and IRE therapies are approved for clinical and practical uses, mild to severe side effects, including pain during ablation,^7^ hemorrhaging, organ failures, infections, blood coagulation necrosis, tissue charring,^8^ and grounding pad burns,^9^ remain potential risks. These demerits mainly result from complicated and bulky ablation systems that typically incorporate bipolar arrays with needle-shaped electrodes that contact targeted areas and grounding pads that interface to the skin surface, as described above. Contact of these needle-shaped electrodes with both targeted tissue and surrounding blood can diminish the efficacy of RFA and IRE due to a higher electrical conductance in the blood compared to the tissues. Furthermore, the lack of biological signal monitoring in RFA and IRE processes may result in ineffective treatment (e.g., due to insufficient power) as well as potential side effects (e.g., due to overheating in RFA). Compared to conventional needle or cuff electrodes for ablation, programmable planar electrode arrays have emerged as versatile biomedical tools with the capability of precisely targeting the area of interests (e.g., tumor tissue), thereby reducing the undesired ablation to adjacent tissues. Furthermore, direct and conformal contact with tissues enhances thermal energy dissipation into the tissues rather than surrounding biofluids.^10^

Materials for RFA and IRE electrodes, that can provide reliable thermal performance and offer opportunities for optical diagnostics and real-time monitoring of ablation processes, are critically important. As such, one of the desired features for an integrated endoscope system is high-optical transparency of the ablation electrodes to enable direct observation and/or further facilitate optical based therapies. In this regard, transparent micro-electro-mechanical systems (MEMS)-based electrodes can be mounted onto the tip of an endoscope to provide minimally invasive ablation treatments while allowing for direct observation. Such devices can significantly reduce the required treatment times and enhance the effectiveness of the therapeutic procedures. For endoscopy and catheter-based ablation systems, common functional materials include metals, alloys, silicon, and emerging 2D carbon-based materials (e.g., graphene, carbon nanotube).^11,12^ For instance, among numerous metallic materials and alloys used in integrated electronics for biomedical devices, gold is a common choice for stimulation electrodes with its sensing capability. As such, gold nanomesh architectures have been employed to spatially map physiological signals in the brain.^13^ Additionally, catheters integrated with a range of gold-based electrodes and sensors (e.g., temperature, impedance, and pressure sensors) can enhance the RFA performance.^6,14^ Nonetheless, due to its intrinsically opaque property, gold is not an ideal material for therapeutic applications that require or benefit from direct optical observation. Furthermore, in optogenetics and light-activated schemes for neuromodulation, semiconductors are preferred as they can offer the capability of interface modulation between electronics and biotissue.^15^ Recent studies suggest 2D materials (e.g., graphene) and metal nanowires as relevant choices for simultaneous tissue ablation and observation. These materials can offer excellent optical transmittance, allowing light from a standard endoscope to pass through active electrodes, thereby enabling the visual assessment of ablation sites.^11^ However, the large surface to volume ratio in 2D materials leads to chemically active surfaces that can cause instability, particularly for applications in biofluid at increased temperatures.

Apart from RF ablation, the integration of electrodes into endoscope systems can also facilitate physiological mapping and stimulations for nerve therapy.^11^ As such, advanced neuromodulations through electrical stimulations of peripheral nervous systems have been demonstrated in improving the recovery of motor neurons and reactivation of muscle contraction to produce movement of paralyzed limbs in patients with spinal cord injuries.^16,17^ Recent studies suggested that neural electrodes with a high-optical transparency can enable the simultaneous combination of physiological stimulation and optical observation/recording, taking advantages of temporal and spatial resolutions in both techniques.^13^ Therefore, the development of transparent and robust electrodes for endoscope systems will provide versatile and powerful tools for the diagnosis and treatment for a wide range of chronical diseases. Nevertheless, there are daunting challenges hindering the development of reliable and robust bioelectronic systems for tissue ablations. Semiconductors, especially those with excellent optical transmittance and long-term stable performance, are suitable materials for such applications. Silicon carbide (SiC) is an emerging bioelectronic material presenting outstanding biocompatibility, functionality, and robustness in biochemical environments.^18−23^ Compared to over 200 types of SiC polytypes, 3C-SiC is the material of choice for the development of microelectromechanical systems (MEMS) devices, owing to its excellent compatibility with Si-based micro/nanofabrication and machining. Previously, we reported an anodic bonding technique that enables the transfer of SiC nanothin films onto an insulator (i.e., 6-in. glass substrates) and is highly suitable for wafer-scale fabrication.^24^ In addition, as the thickness of our 3C-SiC films is typically less than 1 μm, it is practical to pattern transparent SiC microelectrodes using standard dry etching processes in CMOS technology, which is, on the other hand, difficult to implement for bulk materials (e.g., 4H-SiC, 6H-SiC).^25,26^

Utilizing the superior electromechanical and electronic properties of 3C-SiC, herein we develop a robust and transparent planar electronic platform mounted on surgical endoscopes for tissue ablation, with the capability of multiple biological sensing while allowing for direct observation of ablation procedures. The integrated SiC bioelectronic system consists of transparent SiC bioelectrodes for bipolar tissue ablation and multiple biosensors such as those for impedance/touch, viability, temperature, and neuromodulation. Here, we demonstrate applications of SiC electronics in radiofrequency thermal ablation and irreversible electroporation via *in vitro* models, showing fast response, controllable ablation volume, and real-time observation capability. These features highlight the promising potential of SiC integrated electronics for advanced health care and therapeutic applications.

## Results and Discussion

### Structural Configuration

**Figure 1:**
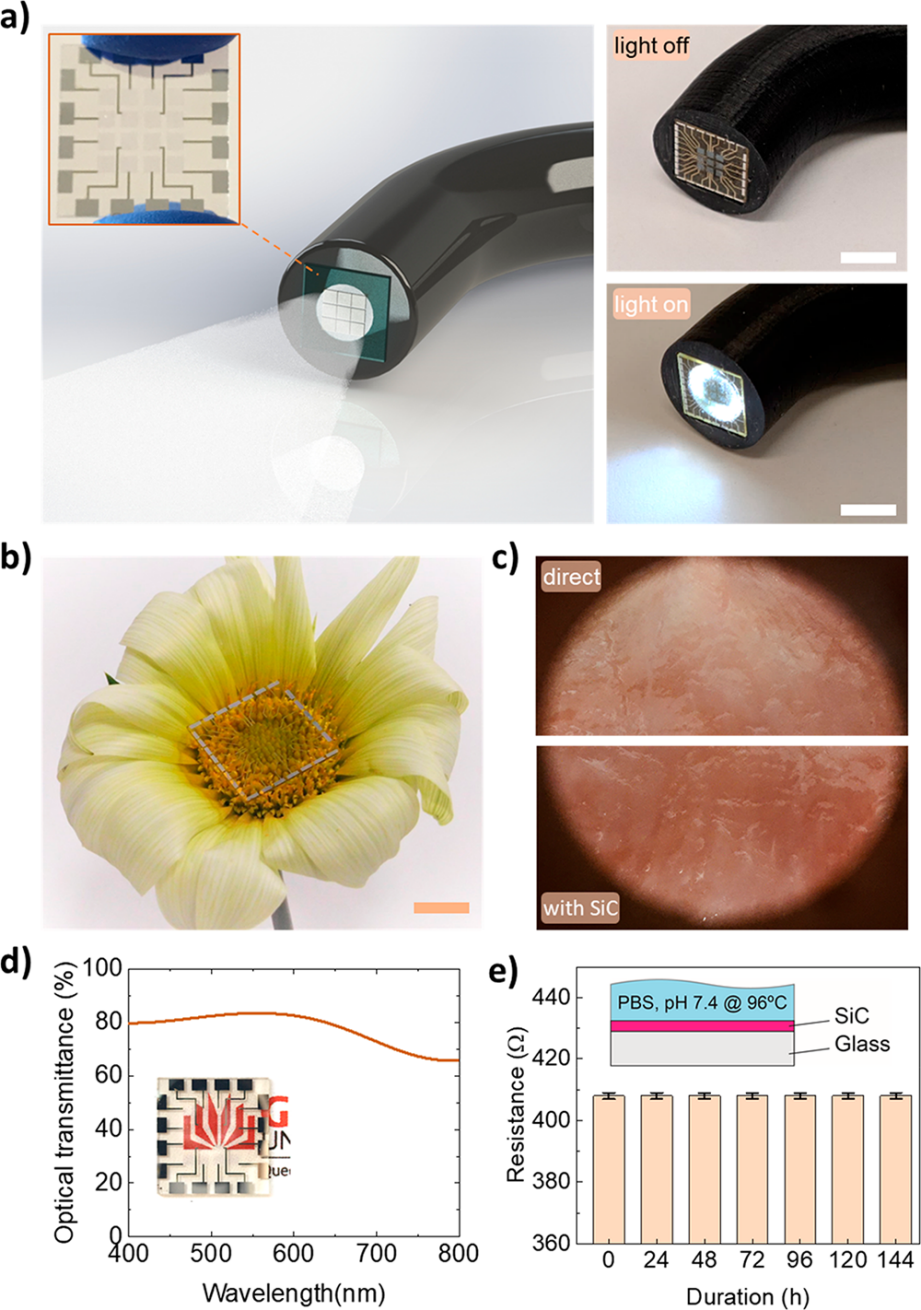
A multifunctional endoscope system integrated with SiC bioelectronics. (a) Left: schematic illustrations of the concept for a multifunctional endoscope system based on transparent SiC bioelectronic devices. Right: Optical images of the transparent SiC integrated on an endoscope when the light is on and off. Scale bar, 5 mm. (b) Optical image of multifunctional and transparent SiC bioelectronics for ablation using an integrated endoscopy. Scale bar, 3 mm. (c) Optical images through SiC electrodes, when in conformal contact with tissues, compared to the direct observation. (d) Optical transmittance measurements with visible wavelengths, showing excellent transparency for observation and allowing for further optical treatments (inset: optical image of transparent SiC bioelectronic chip). (e) Resistance variation of SiC soaking in PBS 1×, 96 °C for up to 144 h.

Figure 1a shows the concept of our biointegrated system, where SiC electronic devices integrate onto the tip of a standard endoscope used in medical diagnostics, typically via the gastrointestinal tract. In the proposed configuration, SiC electrodes are arranged in an array in which each pair of electrodes can be selectively activated to remove the targeted tissue via the bipolar ablation mode (Figure 1a, left, inset). Taking advantage of its transparency in the visible wavelength, light can pass though SiC electrodes and sensors to allow assessments and visualizations of areas of interest for ablation (Figure 1a, right). Prior to the ablation procedures, these SiC electrodes can function as an impedance sensor to confirm contact between the electrodes and tissue, which is critically important to treatment outcomes. Once these electrodes are in the desired position, AC electrical signals are applied in the radio frequency (RF) range for tissue removal. Local temperature can be continuously monitored during the ablation process. Depending on the patterns of applied RF power, these SiC electrodes offer different routes of thermal ablation or electroporation modes to effectively remove disordered biotissue. Once the energy-based treatment is accomplished, the SiC electrodes function as viability sensors to verify the efficacy of the ablation procedure. Figure 1b,c demonstrate the excellent optical transparency of the SiC bioelectronic platform. For demonstration, an SoG chip was placed in the center of a flower (Figure 1b), and clear observation is preserved when attaching the chip onto the tip of an endoscope camera (Figure 1c). Figure 1d plots the optical transmittance of SoG electrodes measured by a spectrometer (i.e., Nanospec AFT 210). The data show a high average transmittance of approximately 75% over the visible spectrum in our SiC platform (wavelengths from 400 to 800 nm), much larger than most metallic materials with a similar thickness. This excellent optical transparency is realized due to the high energy band gap of 2.3 eV in a cubic SiC crystal, corresponding to a UV range of absorption (sub-400 nm wavelength).

To assess the long-term stability of the SoG in biofluid, hydrolysis measurements were performed by soaking SiC electrodes in a phosphate-buffered saline (PBS) 1× solution at 96 °C for 144 h. As shown in Figure 1e, a consistent electrical conductivity in SiC materials over a 144-h period of testing time proves the feasibility of SiC electrodes for RFA applications which are associated with high temperatures and increased corrosion. The thickness variation under the same hydrolysis conditions was tested and obtained to be negligible for a duration of 14 days (Supporting Information, Figure S1a). Accordingly, the degradation and variation in the SiC electrical conductance and thickness were found to be negligible (i.e., less than 1% after 14 days in PBS 1× at 96 °C; Figure 1e and Supporting Information, Figure S1a). We further benchmarked the SiC electrodes with one of the most common materials for electrodes in lab-on-chip applications and neuron modulation, indium tin oxide (ITO).^27^ It offers excellent optical transparency of over 80% and high electrical conductivity,^28^ therefore, highly suitable for neuron modulation or electrical stimulation applications that require real time observation. The experimental data showed that the conductivity of the ITO film varied significantly (up to 250%) after 5 days soaking in the solution (see Supporting Information, Figure S1b). This significant change in electrical conductivity of ITO is attributed to a combination of the hydrolysis reaction, the absorption of water molecules, and the diffusion of ions from the biofluid into the film.^29^ Apart from ITO, a transparent graphene/silver nanowire platform has been recently proposed for integrated RF endoscopy via the gastrointestinal tract.^11^ However, due to their highly active surface state, 2D materials generally suffer significant surface oxidation and water penetration, leading to unstable performance for long-term use in biofluids.^30,31^ Moreover, potential drawback of using nanowires is the limited adhesion to their substrates due to the weak van der Waals forces, resulted from associated synthesis techniques that are mainly based on sonification or drop casting.^32^ Therefore, the use of those materials for RFA typically could lead to the limitation of operational lifetime, short ablations, and the delamination of the functioning elements from the substrate due to the high voltage and relatively high temperatures during RFA procedures. In contrast, using transparent SoG for tissue ablation gives significant advantages for reliable treatments with minimized cytotoxicity and the release of byproducts to the body. As SiC is a semiconducting material by nature, it offers a modulable bioelectronic–biotissue interface through external stimuli (e.g., thermoresistive effect for temperature sensors, electro-impedance for viable or contact sensors). Incorporating multimodal components such as RF electrodes, impedance sensors, and temperature sensors in a monolithic SiC electronic platform provides a versatile tool for tissue ablation applications. The consistent electrical conductivity as well as film thickness in 3C-SoG indicate its potential for long-term reliable RF ablation, where material stability at high temperature and high humidity is critically important.

### Multifunctional SiC Biological Sensors

**Figure 2:**
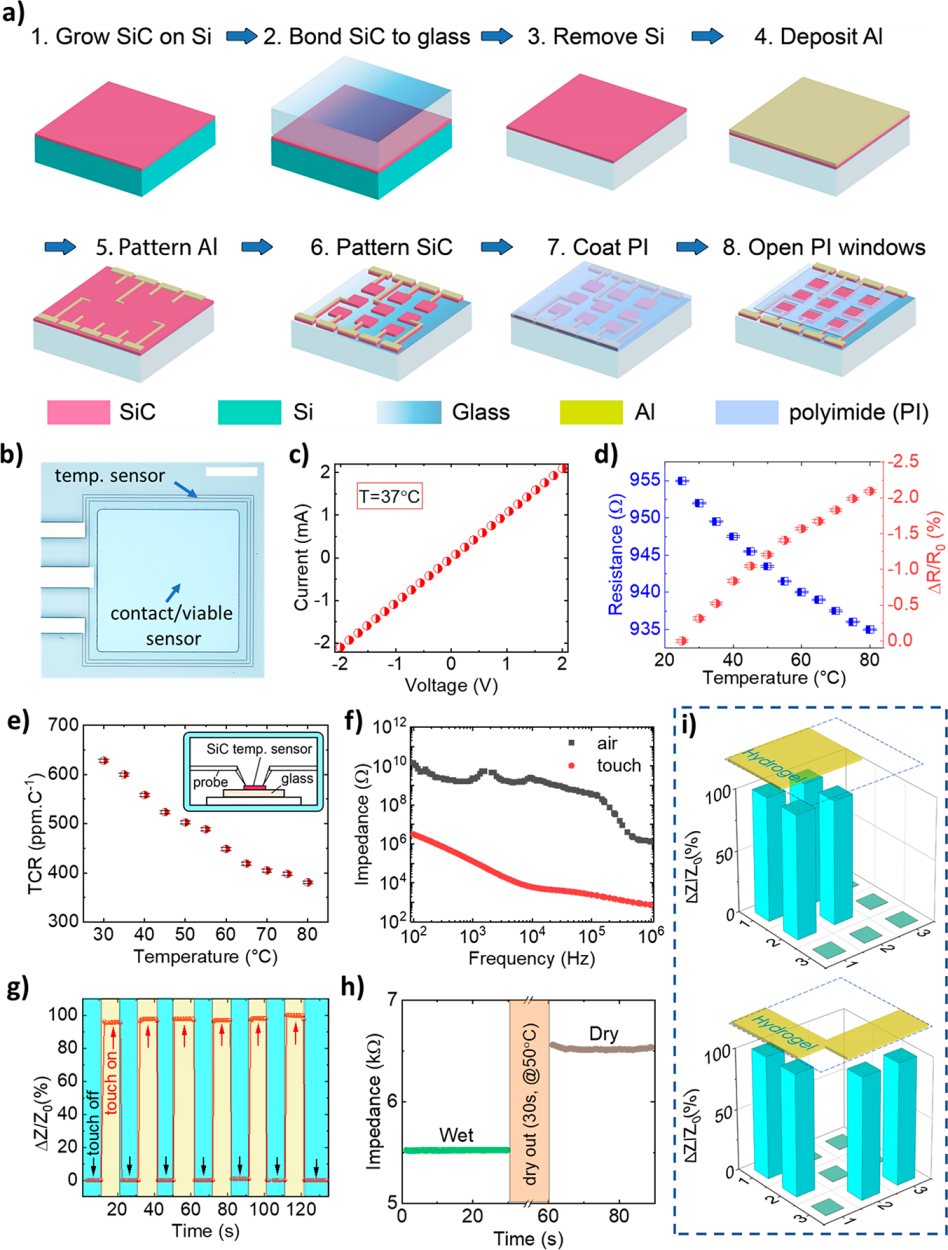
Transparent bioelectronics for multifunctional sensing. (a) Fabrication process flow for integrated 3C-SiC electrodes: (1) grow SiC on Si wafer, (2) bond SiC to glass, (3) remove Si, (4) deposit metal contact (Al), (5) pattern metal, (6) pattern SiC, (7) coat polyimide, (8) open PI to expose SiC electrodes, (b) Optical image of temperature and contact/viable sensors. Scale bar: 300 μm. Characterization of (c) current–voltage characteristic of SiC temperature sensor, (d) resistance and relative change of resistance upon increasing temperature, (e) temperature coefficient of resistance from 25 to 80 °C. Inset: setup of temperature measurement. (f) Impedance spectra of the electrode when in contact and off-contact with hydrogel. (g) Touch sensor and (h) viability sensor by impedance measurements. (i) Spatially mapping for contact sensors in a 3 × 3 array.

Figure 2a presents the engineering approach to form integrated SiC bioelectronic systems starting with the growth process of single crystalline SiC on a Si wafer. We epitaxially deposited ultrathin SiC films on Si wafers using an alternating supply epitaxy with silane and propene.^33^ The crystallography orientation, crystallinity, and surface roughness of the films were characterized using TEM and SAED measurement (see Supporting Information, Figures S2 and S3). The film thickness was found to be approximately 200 nm using NanoSpec AFT 210. The surface morphology of the SiC films was measured using atomic force microscopy (AFM; i.e., Park Systems NX20), showing a surface roughness of less than 4 nm (see Supporting Information, Figure S3). We then transferred the as-grown SiC films onto a highly transparent glass substrate (e.g., 6-in. Pyrex glass wafer, sourced from Semiwafer) using an anodic bonding process.^24^ A combination of plasma surface treatment and mechanical pressurization resulted in a good adhesion between SiC and glass of over 20 MPa,^24^ enabling subsequent micro/nano-micromachining steps to form SiC electronics (see Supporting Information, section 2). Next, micromachining processes such as lithography, metal sputtering, and inductive coupled plasma (ICP) etching were applied to fabricate arrays of SiC electrodes, metal interconnects (i.e., aluminum), and electrical encapsulation (i.e., polyimide (PI)). Here, the PI layer ensures that only SiC areas are in contact with biological targets while all metal traces are isolated from the surrounding biofluid environment. The SiC electrodes and sensors were then diced into individual chips using a dicing saw to fit the size of medical endoscopes. The growth process, wafer bonding technique, and microlithography procedures are suitable for large scale wafer development (i.e., 6-in. wafer in the present work), and thereby they are highly compatible with industrial MEMS and complementary metal-oxide-semiconductor (CMOS) technologies. This feature represents an advantage over alternative approaches based on 2D semiconductors, where large scale material and device development remain a challenge. Utilizing the fabricated platform, we demonstrated a range of biophysical sensors of relevance to the bipolar RFA process, including temperature, impedance (i.e., touch), and viability sensors (Figure 2b–i).

### Temperature Sensing

Monitoring temperature variations is of particular importance to ensure optimal electrical stimuli, possibly in a closed-loop control system to enhance RFA outcomes. For instance, RFA conducted at a temperature range of 40–45 °C only induces irreversible tissue damage after relatively long exposure times (30 to 60 min).^34^ The required exposure time decreases exponentially as the ablation temperature exceeds 60 °C, at which the protein denaturation can occur and lead to coagulative necrosis.^35^ To precisely measure the heat generated from ablation sites, we constructed SiC temperature sensors in a U-shaped configuration surrounding the periphery of SiC ablation electrodes (at a distance of 30 μm, Figure 2b). The linear current–voltage characteristic in an operating voltage range of −2 V to +2 V indicates a good ohmic characteristic of the sensor (Figure 2c). Figure 2d,e plot the response of the SiC sensors for the operational temperature range in typical RFA from 25 to 80 °C. The significant resistance change over the tested temperature range indicates that the SiC material is a good candidate for built-in temperature sensing elements. In highly doped n-type SiC, electrical resistance typically decreases with the increasing temperature due to the dominance of thermally activated carrier density over the decrease in electron mobility caused by phonon scattering. As such, the majority of charge carriers are ionized near room temperature, while the phonon scattering only becomes significant at higher temperatures. The electrical conductivity change with temperature is represented by σ ∼ *T*^–α^ exp(−(*E*_a_/*k*_B_*T*)), where *E*_a_, *k*_B_, and *T* are the activation energy, Boltzmann constant, and ambient temperature, respectively; α is a constant representing the scattering of charge carriers. Subsequently, the thermal sensitivity of SiC can be quantified by the temperature coefficient of resistance, TCR = (Δ*R*/*R*)/Δ*T*. The SiC temperature sensors were calibrated in a temperature control chamber with a K-type thermal couple (i.e., FLUKE 714B) as the reference sensor. Details of the calibration procedure can be found in the Supporting Information, section 3 and Figure S4. The TCR of SoG was found to be approximately 628 ppm C^–1^ at 30 °C and 380 ppm C^–1^ at 80 °C, indicating a good sensitivity for temperature monitoring (Figure 2e).

### Impedance Sensors for Tissue Contact Detection

Figure 2f–i show the characterization of integrated SiC impedance sensors for the detection of conformal contact between ablation electrodes and tissues as well as measurement of tissue viability. The sensor measures the electrical impedance between a pair of SiC electrodes selectively activated for RFA and IRE. In the noncontact mode, the impedance is dominated by the oxide capacitance between the two SiC electrodes. When the electrodes are in contact with tissues, an additional electrical conduction path is formed between electrode/tissue/electrode that reduces the total impedance.^6^ To verify this sensing concept for integrated SoG sensors, we periodically placed the sensors ON- and OFF-contact with a hydrogel surface (i.e., a phantom skin with a mixture of 2 wt % agar, 98 wt % PBS 0.1 M, Supporting Information, section 4) and monitored the impedance change in real time using a programmable LCR meter (i.e., HP4284A). Figure 2f plots the impedance (*Z*) in a frequency range of 100 Hz to 1 MHz, showing a decrease in the impedance with increasing the measured frequency, which is attributed to the capacitance component (*Z*_c_ = 1/*j*2π*fC*). We also observed a marked change in the impedance under ON- and OFF-contact modes, clearly differentiating the contacting state of the sensors with the targeted tissue (i.e., the contact impedance is approximately 4 orders of magnitude lower than that of OFF-contact). For instance, at a frequency at 10 kHz, the sensors exhibit excellent repeatability over several ON- and OFF-touching cycles with a fast response time of about 800 ms (Figure 2g). Utilizing this impedance sensing concept, it is possible to validate the effectiveness of RFA. In particular, tissues typically dehydrate after RFA treatment. As impedance highly depends on water concentration, impedance sensors offer a simple approach to validate the viability of tissue post-RFA. We demonstrate this application in Figure 2h, where SiC impedance sensors can detect hydrogel phantom tissues with high and low water concentrations. The hydrogel was placed on a hot plate at a temperature of 50 °C for 30 s to reduce its water content. A significant change of 20% in the impedance of the hydrogel before and after being dried out confirmed the feasibility of our SiC sensors for viability assessment. Furthermore, simultaneously measuring impedance at different locations enables mapping of tissue surface morphology as well as contact areas. For instance, the SiC sensors can detect the phantom tissues with different shapes such as squared and L-shaped arrangements, Figure 2i.

### Integrated SiC Bioelectronics for Tissue Ablations

#### Radio Frequency Thermal Ablation

Applying a high frequency (>10 kHz) alternating current to tissue can increase the temperature while only causing minimal muscle contraction and pain.^5^ The main energy-transfer mechanisms for the heating are (i) conduction losses associated with resistance to charge carrier flow (i.e., resistive heating) and (ii) dielectric losses due to dissipated heating from molecular rotations in an alternating electric field. The dielectric losses are negligible when the alternating frequency is below 1 MHz,^36^ and the heating phenomenon can be simplified into solely the resistive heating. Radio frequency ablation is typically conducted at a preferred frequency between 400 kHz and 500 kHz, which is optimal for molecular frictional heating and for controlling energy transmission through the tissue to avoid unnecessary radiation.^37^ Generally, the electric field strength directly causes heating in the vicinity of the electrodes. Thermal conduction assists in transferring thermal energy to regions further from the electrodes.^38^ Subsequently, the thermal energy penetrating into the depth of the tissue by heat transfer can facilitate the removal of tumors for specific treatments. Theoretical equations of electric field distribution and heat transfer can be employed to model the radio frequency ablation as^38^
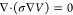
1
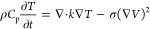
2in which σ, ρ, *C*_p_, *T*, *k*, and *V* are electrical conductivity (S m^–1^), mass density (kg m^–3^), specific heat capacity, temperature, thermal conductivity of the ablated tissue, and applied voltage, respectively. Equation 1 presents the Laplace solution defining the potential distribution in electrical based ablations, while eq 2 describes the relationship between tissue temperature change versus thermal conduction and resistive heating power. Neglecting the heat loss to its host substrate (i.e., Pyrex glass), one can find the solution for the heat transfer in bipolar electrodes for tissue temperature change, Δ*T*, as a function of tissue electrical conductivity, electric potential, and electrode dimensional parameters.

**Figure 3:**
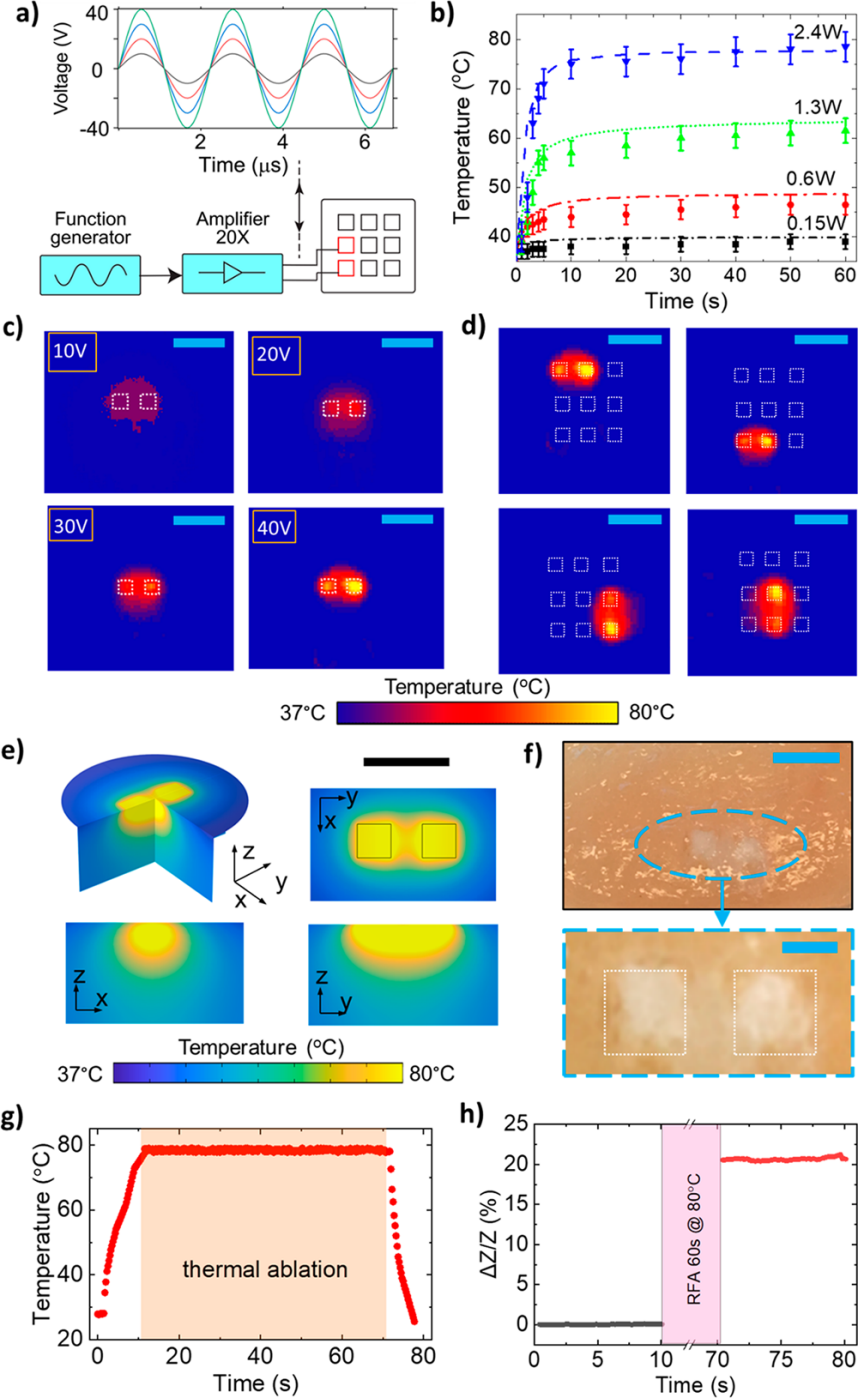
(a) Measurement apparatus for RFA at radiofrequency AC. (b) Transient temperature (max) with an applied power of 0.15, 0.6, 1.3, and 2.4 W. Thermal images of RFA with an in vitro model of ionic hydrogel (2 wt % agar, 98 wt % 0.1 M phosphate-buffered saline (PBS)) at (c) varying applied RFA voltages of 10, 20, 30, and 40 V. Scale bar, 4 mm. (d) Selection of ablation electrodes within the array at an applied voltage of 40 V. Scale bar, 4 mm. (e) FEA modeling showing spatial temperature profile distribution at 40 V. The maximum temperature is in the electrodes area and can reach 80 °C. Scale bar, 3 mm. (f) Chicken breast lesion after 10 s RFA, indicated in white color; SiC electrodes outlined in white lines. Scale bar: top, 3 mm; bottom, 1 mm. (g) Real-time temperature during RFA process, duration for ablation is approximately 60 s. (h) Viability test by impedance measurement before and after thermal ablation confirming the targeted tissue ablated.

The sinusoidal AC input was fixed at a frequency of 450 kHz throughout the RFA experiment, Figure 3a. The input RF voltages (10 to 40 V), corresponding to input power from 0.15 to 2.4 W, were applied selectively to a pair of adjacent SiC electrodes with conformal contact to the hydrogel. An infrared (IR) thermal camera (Xenic Obi 384) with a spatial solution of 25 μm was employed to visualize the temperature distribution over the ablated area. The transient maximum temperature at the ablated regions was fit between analyses and experiments showing a fast-rising temperature response upon application of the AC voltages. In the sinusoidal voltage range from 10 to 40 V (*V*_0_), the ablated region reached the desired temperature within less than 10 s, then saturated due to reaching the thermal equilibrium state (Figure 3b). Figure 3c presents the temperature distribution under applied voltages of 10, 20, 30, and 40 V, respectively. Evidently, the temperature profile was clearly visualized around the area that connects the bipolar electrode pair, where the maximum temperature was observed at the center of each electrode. Increasing the applied voltage leads to significant increases in the ablated tissue temperature. Consequently, the temperature can reach 80 °C at an RF voltage of 40 V that is very sufficient for the tissue thermal ablation. This high temperature heating capability offers a significant benefit for RFA, as a typical procedure at a temperature above 60 °C can effectively reduce the ablation time to less than 1 min.^39^ Furthermore, by selectively activating pairs of SiC electrodes at different locations, we can precisely apply RFA to desired regions without the need for maneuvering the whole array (Figure 3d). This result suggests a promising possibility for a multiplex, programmable ablation approach to target tissues or tumors with complex configurations and morphologies.

It is worthy to note that while SiC offers superior properties of thermal/corrosive tolerance along with transparency over metals, it only requires a similar range of power consumption for RF ablation. In particular, the RFA is caused by the Joule heating effect at the electrode/tissue interface under an AC current. The constructing material for electrodes typically exhibits a much higher electrical conductivity than that of biotissue; therefore, the power consumption mainly depends on the electrical properties of biotissue. The power consumption of our SiC-based system was estimated on the basis of the transconductance current and applied voltage, which was found to be in a range from 0.15 to 2.4 W, associated with an RFA voltage from 10 to 40 V. The data suggest that, owing to the good electrical conductivity (∼250 S m^–1^), the highly doped 3C-SiC devices can function with a relatively small operating power of several watts, comparable with other systems (Table 1).^11^

**Table 1:** Integrated Material Systems for Radio Frequency Ablations

material/platform	power	frequency	applications	transparency	ground pad required?
DLM@PLGA nanocapsules^43^	3 W		ex vivo tissue	low	no
iron oxide nanoparticles^44^	5 W	513 kHz	tumor tissue	no	no
Ag needle electrodes^45^	15 W	500 kHz	liver tumor	no	no
carboxylated graphene^46^	25 W	<1 MHz	cancer cells	no	yes
calcium phosphate nanoparticle^47^	10 W	450 kHz	tumor tissue	no	no
Au thin films^14^	0.8 W	400–500 kHz	cardiac tissue	low	no
graphene + Ag nanowires^11^		500 kHz	colon cancer treatment	**high**	no
Au thin film^6^	0.4 W	400 kHz	ex vivo tissue	low	no
SoG [this work]	2.4 W	450 kHz	ex vivo tissue	**high**	no

To theoretically verify the experimental results, we developed an finite element analysis (FEA) model using COMSOL Multiphysics to estimate the ablation effect of the SiC electrodes (see Supporting Information, section 5 and Figure S5). The model incorporates the electric field distribution and bioheat equation, from which the solution is derived for the applied voltage to achieve the adequate temperature for the tissue termination. As such, it is also possible to obtain the ablated region associated with the spatial temperature distribution. Figure 3e shows that, by applying a voltage of 40 V (equivalent to an applied power of 2.4 W) at a frequency of 450 kHz to a pair of neighboring electrodes, a significant temperature increase to 80 °C was achieved. This highest temperature rise occurs in the SiC electrodes area, and gradually reduces when going further away from the electrodes. These results from the FEA model are consistent with the obtained experimental data in which, at 2.4 W of input power, the temperature rises to an adequate value of nearly 80 °C in approximately 10 s and is then saturated due to thermal equilibrium, Figure 3b. There is a possibility that the target lesion region can be adjusted by varying the electrode geometry and spacing. The ablated region of the FEA model has a similar shape to that of the phantom tissue in the experiments. Additionally, the scaling law involving the tissue temperature distribution (spatial ablated region), electrode sizes *L*, and electrode spacing *D*, in the steady stage of RFA was also investigated. Accordingly, the local temperature rise is proportional to the thermal conductivity and square of applied voltage, Δ*T* ∼ σ*V*^2^. At a given AC voltage, Δ*T* is a function of dimensionless parameters (*L*/*D*, *x*_*i*_/*D*, where *x*_*i*_ is spatial coordinates of the local tissue being ablated, Supporting Information, section 5).

We further demonstrated the RFA capability in transparent SiC devices on an animal tissue model (i.e., chicken breast tissue). The tissue was cleaned using a saline solution (0.9% NaCl), which is a standard clinical cleaning procedure prior to RFA.^40^ A sinusoidal voltage of 40 V was then continuously applied for 60 s to a pair of bipolar SiC electrodes which were brought into contact with the tissue. Figure 3f presents photos of the tissue post RFA, showing a clear tissue ablation at the targeted sites. The effective tissue ablation is also attributed to the excellent electrical insulation of the glass substrate, preventing electric current leakage from SiC electrodes into the substrate during the application of RF power. The temperature was monitored during the ablation process and showed a fast increase to reach 80 °C under 10 s then remained relatively stable for approximately 60 s of a thermal ablation procedure (Figure 3g). The temperature then quickly decreased upon the completion of the RFA. This indicates the fast and effective ablation using SoG electrodes. The impedance of the ablated tissue was confirmed by *in situ* measurements of the impedance of the ablated tissues before and after the RFA with a noticeable change of above 20% at a frequency of 100 kHz at the completion of the ablation, Figure 3h. This is attributed to the dehydration and/or carbonization of the ablated tissue, which is in good agreement with those reported in the literature.^11,41^

We further conduct an *in vitro* RF ablation experiment on an animal tissue (i.e., rat heart tissue) to demonstrate the multifunctionality of the SiC system. As shown in Figure S6, the thermoresistive effect in the integrated SiC sensors enabled real-time monitoring of the temperature profile during the RF ablation procedure. The results also indicate that SiC electrodes can deliver a sufficiently high temperature of 80 °C at a relatively small power of approximately 2 W, which is applicable for thermal-based lesion removal. As well as clinical applications, the optical transparency and the compatibility with standard micromachining offer the SoG platform attractive features for tissue engineering applications. Specifically, the utilization of smart electronics with multimodal sensing capability advances fundamental biological and clinical studies of organs on a chip. As such, the need for live animal tests can be substituted by regenerated on-chip human tissues, yielding more relevant results for tissue engineering.^42^ Our *in vitro* experiments using the transparent SoG devices for observation of cell response to thermal stimuli revealed the promise of this platform for on-chip sensing and monitoring during tissue ablation.

#### Irreversible Electroporation

**Figure 4:**
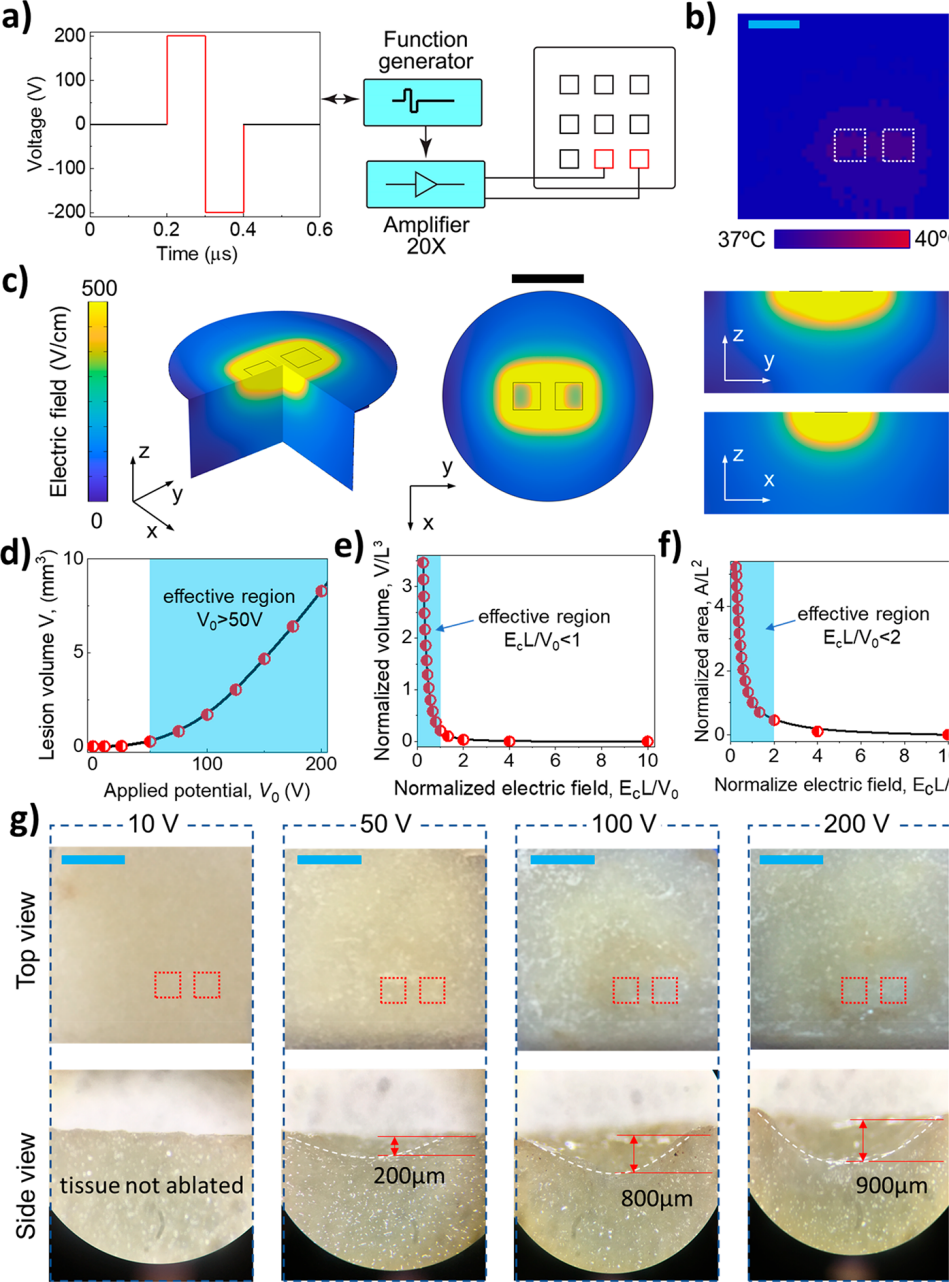
Irreversible electroporation. (a) Schematic illustration of the IRE procedure. (b) Thermal image of the temperature distribution during IRE at 1 Hz (with a temperature increment of ∼1 °C) on the ionic hydrogel; white dashed squares in the bottom right indicate the positions of the SiC electrodes. (c) FEA results for the spatial electric field distributions during IRE. Scale bar, 3 mm. Scaling effect analyses of IRE: (d) Effective lesion volume versus applied voltage. (e) Normalized lesion volume and (f) normalized lesion area versus normalized electric field strength. (g) Optical images of lesions created on a vegetable model (potato) with IRE (1 Hz, 30 s); red dashed squares illustrate the positions of the SiC electrode pairs for IRE. Scale bar, 4 mm.

As an alternative to the RFA technique, irreversible electroporation (IRE) is an ablation method that employs a nonthermal technique for minimally invasive tumor ablations.^48,49^ The main advantage of this method is that IRE causes permanent tissue damage or cell lysis by an electric field from microsecond pulses of high direct currents.^34^ Under a sufficiently high electric field, the permeability of cell membranes increases. At a certain point, membranes are opened, creating irreversible pores and leading to cell lysis by the dielectric breakdown phenomenon.^50,51^ Typically, IRE treatments require an electrical field of approximately 500 V cm^–1^ and a number of short pulses of 100 μs.^34,52^ This short pulse is considered faster than the thermal response time in standard microelectrodes or microheaters, thereby preventing a significant increase in the temperature of the tissue. Subsequently, the IRE correlates with the electric field distribution governed by eq 1 without the bioheat transfer as occurred in the RFA method. We experimentally verified this hypothesis by conducting IRE on a hydrogel phantom tissue under a voltage pulse of 100 μs with an amplitude of up to 200 V at a frequency of 1 Hz over a duration of 30 s (Figure 4a). Using an IR camera to map the temperature profile around the electrode area, we did not observe any significant temperature increase in the phantom tissue (less than 2 °C increase in the ablated tissue temperature; Figure 4b). This result implies that in the IRE method, tissue cell lysis occurs as a result of a high electric field rather than due to the temperature rise as in the RFA technique.

Using the FEA method, we developed a simulation model to investigate the electric field distribution under biphasic voltages applied to SiC electrodes, and a phantom tissue was used as the modeled tissue (see Supporting Information, section 7 and Figures S7–S9). In our FEA simulations, different voltages of 10, 50, 100, and 200 V were applied with a pulse length of 100 μs and a frequency of 1 Hz. Figure 4c shows the electric field strength distribution for the applied voltage of 200 V, which mainly concentrates on the electrode area and spreads to its vicinity in the 3D space (i.e., around and under the electrodes). Accordingly, the highest electric field strength (∼500 V cm^–1^) is in the vicinity of a pair of electrodes expanding to the surrounding areas following the scaling law.

One of the most critical requirements of IRE is the matching of electrical potential with the targeted lesion volume. In IRE, the lesion is formed in a region with a generated electric field strength greater than a threshold (e.g., 500 Vcm^–1^). In the case of biplanar electrodes, the electrode spacing is an important parameter which dictates the electric field distribution and the depth of the formed lesions.^53^ As such, at a given voltage and electrode size, a smaller spacing typically generates deeper lesions, which is consistent with the scaling relationship as discussed below. The correlation between the electric field strength with the dimensions of IRE electrodes follows the scaling law detailed in the Supporting Information, section 7.

Figure 4d shows this relationship in which the lesion volume is negligibly changed when the applied voltage is below 25 V; then it significantly increased when the voltage ranged from 50 to 200 V. This is a useful indication for the effective working voltage for the present SiC bioelectrodes in the IRE. Scaling effects were analyzed for lesion volume versus applied voltage magnitude and lesion volume versus normalized electric field strength, Figure 4d and e, respectively. As such, the lesion volume started to increase significantly when the applied peak-to-peak voltage reached 50 V, Figure 4d. Furthermore, the normalized lesion volume (*V/L*^3^) increases sharply when the normalized electric field strength *E*_c_*L*/*V*_0_ < 1 (here, *E*_c_ is the critical electrical intensity (500 V cm^–1^), which causes tissue damage, in which *L* is the distance between adjacent electrodes and *V*_0_ is the applied voltage). The effective ablation volume was diminished when *E*_c_*L*/*V*_0_ significantly exceeded 1 (e.g., with a relatively low voltage or with a long distance between activated electrodes). Figure 4f shows the relationship between the effective lesion area versus normalized electric field strength in FEA simulations. A similar trend was observed where a higher applied voltage (*E*_c_*L*/*V*_0_ < 2) results in a larger normalized ablation area (*A*/*L*^2^).

To demonstrate IRE using the SoG electrodes, we conducted an *in vitro* experiment using a vegetable tissue model. The potato tissue was chosen for the IRE experiment due to the ease of the visualization of tissue damage through a natural oxidation process.^54^ As such, after IRE, the ablated potato tissue changed its color as well as volume from the released intracellular enzymes. The enzymes react with oxygen (in air), leading to the polymerization which changes the color and volume of the ablated tissue. We applied biphasic voltage pulses with magnitudes of 10, 50, 100, and 200 V at a 100 μs pulse width and a frequency of 1 Hz for 30 s. All samples were then left under ambient conditions for 24 h for subsequent optical analyses. Figure 4g shows cross-sectional images of potato samples after the IRE ablation at different voltages. At 10 V, no significant surface change was observed, indicating that the applied electric field strength is insufficient to cause tissue to be ablated. A change in color and ablated volume in potato tissues started to appear at voltages above 50 V. The ablation area observed from the top view expanded significantly when increasing the voltage pulse to 100 and 200 V. The ablated lesion depths from the IRE at 50, 100, and 200 V were found to be approximately 200 μm, 800 μm, and 900 μm, respectively. These experimental results of ablated region and depths were in good agreement with the modeled electric field spatial distribution in FEA (Figure 2c). Our results prove the potential of SiC devices for deep-tissue ablation using IRE as a controllable and low power consumption technique without unwanted tissue heating.

#### *In Vitro* Cell Lysis and *in Vivo* Nerve Stimulation

**Figure 5:**
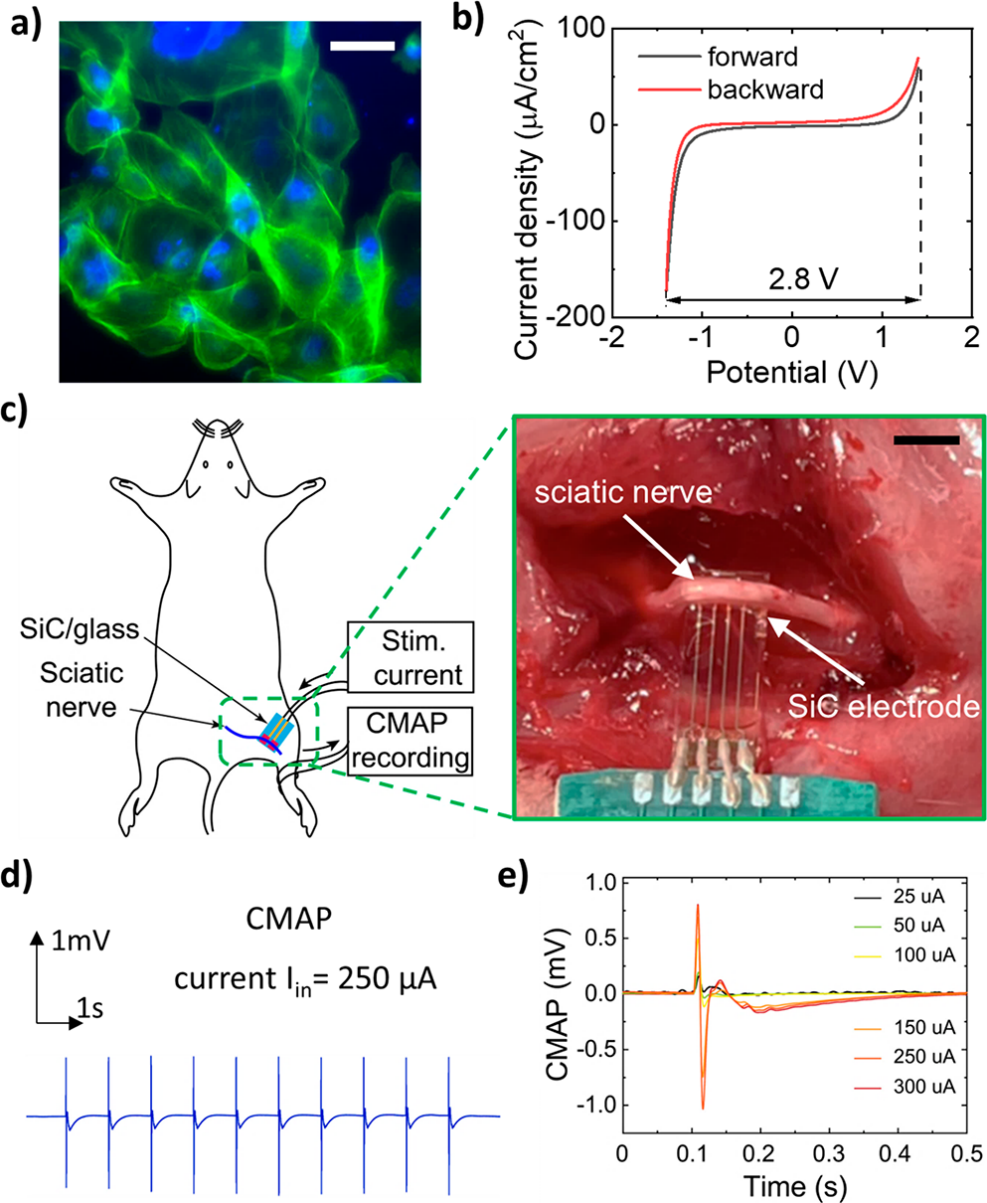
Demonstration of in vitro cellular assays and in vivo muscle stimulation using an SoG electrode: (a) Fluorescence image of cell proliferation: actin (green) and nucleus (blue) show their morphology for attachment and spreading. Scale bar, 20 μm. (b) Cyclic voltammetry scan of SiC electrode in 0.1 M PBS (pH = 7.4) at a scan rate of 0.1 V s^–1^, and large potential windows of 2.8 V obtained for SiC bioelectrodes. (c) Left: Schematic sketch for stimulation of the sciatic nerve in anaesthetized rat, not to scale. Right: photograph of SoG electrode contacted with a sciatic nerve, scale bar, 5 mm. (d) Continuous CMAP recorded from transcutaneous surface electrode at applied current of 250 μA at 1 Hz. (e) CMAP with different applied stimulating currents from 25 μA to 300 μA using the SoG electrodes.

There is great demand for the development of optically transparent materials that enable simultaneous therapeutic treatment and observation for both clinical applications and fundamental research.^55^ Constructing materials with optical transparency allows for highly integrated multifunctional components (e.g., ablated electrodes and sensors) on the tip of endoscopes without blocking the field of view of the camera.^11,13^ Here, using an *in vitro* approach, we further demonstrate that SiC has an excellent optical transparency of approximately 80% in the visible wavelength. This enables real-time and continuous observation of energy-based thermolysis at cell levels. Prior to the on-chip studies, we investigated the biocompatibility of the transparent SoG platform using an *in vitro* cell growth with MDA-MB-231 cells (Sigma-Aldrich), an epithelial breast cancer cell line. The favorable optical transparency in the visible wavelengths allows for the direct observation of cell growth and proliferation using a conventional inverted microscope (see the Methods section). The cells were seeded and cultured on the SoG and a control substrate for set time points; then their proliferation on the SoG surface was directly observed (Figure 5a). To examine the cell proliferation in SiC and a control sample, cell numbers were counted, showing comparable proliferations between SiC and the control substrate for either cell direct contact or on an eluate (see Supporting Information, Figure S10). We then employed an *in vitro* approach to demonstrate the use of optical transparency in SiC for real time observation of thermal ablation where cells were grown on the top surface of electrodes while an inverted microscope was used to observe the cell lysis process (e.g., Petri dishes; Figure 5a). The transient temperature of the electrode was monitored utilizing the thermoresistive effect of integrated SiC temperature sensors described above. The temperature profile was then correlated to the cell response to thermal ablation.

At a relatively low power (0.15 W), which corresponds to a temperature increase from 25 °C to 30 °C, cells retained their adhesion to the SiC electrode. At 2.2 W, the cells were gradually ablated, although a cluster of cells remained viable after 1 min. Nevertheless, increasing power up to 3.5 W led to the completed ablation of cells from the electrode area (Figure S11a, inset). The transient response indicates that the cells were completely removed after less than 10 s at an applied power of 3.5 W. The high thermal conductivity of SiC is considered to contribute to this fast thermal response of the lysis process. Our observation also suggests that as the heat was mainly constrained around the activated electrodes, cells seeded on outer areas of the SiC ablation electrodes retained good adhesion to the substrate (Figure S11a, inset). This demonstrated the possibility for selective thermal ablation using programmable electrode arrays. The capability of real time observation of RF ablation provides valuable insight into improving the safety and treatment outcomes and underpins fundamental research through lab-on-chip or organ-on-chip platforms.

In addition to tissue ablation, endoscope systems capable of physiological mapping and stimulation electrodes can also provide powerful tools for nerve therapy (e.g., vagus nerve stimulation) or cardiac electrophysiology (e.g., defining abnormal areas or pacing).^56^ Our SoG electronic platform, with its low impedance and wide electrochemical windows, is presented as a promising candidate for nerve stimulation. We validated the potential of SiC thin films for endoscopy-based nerve stimulation using *in vivo* experiments on an animal model (i.e., a rat model). It is imperative to investigate the electrochemical impedance spectra (EIS) of SiC as active electrodes in a simulated biofluid (PBS 1× pH 7.4). The measurement was conducted by scanning the frequency from 80 Hz to 100 kHz, using a CH Instruments CHI 660E. The impedance of SiC electrodes significantly decreased at higher frequencies and then stayed below 1 kΩ when the frequency was above 10 kHz (see the Supporting Information, Figure S11b). This relatively low electro-impedance of the SiC electrodes is preferred for ideal Faradaic interfaces in nerve stimulations as it allows for large capacitive current injection while minimizing degradation due to the Faradaic effect.^57^ This desired feature is attributed to the fact that the impedance of SiC can be controlled by increasing impurity concentration in the SiC growth process. Furthermore, the potential window of the SiC electrodes was measured by cyclic voltammetry (CV) in 1× PBS. As such, there is a wide potential window of 2.8 V, without any redox peak (Figure 5b). This value is one of the highest reported for semiconductor-based materials (e.g., larger than that of boron-doped diamond and most metals such Ag, Au, Pt^58^), presenting exciting opportunities for bioelectronic applications. The wide potential window also indicated the enhanced safety for nerve stimulation without undesired electrochemical reactions with water for long-term operations, as well as the capability of analyte detection^59^ without the interference of the background current caused by the reduction/oxidation of water. Cyclic voltammetry measurement using our SoG electrodes indicated that water electrolysis did not occur at potentials between −1.4 and +1.4 V (Figure 5b). The current was shifted to more negative at potentials below −1.3 V, attributed to the oxidation reduction.^60^ We further assessed the charge injection capacity at the potential window limit of the transient voltage corresponding to an applied biphasic current with an amplitude of 300 μA, with a 100 μs pulse width. In the charge injection, the electrode capacitance is derived as *C* = *I*/(d*V*/d*t*), where *I* is the pulse amplitude (A), and d*V*/d*t* is the electrode polarization given by the linear aggression from the voltage excursion slope (Figure S12). Accordingly, the charge injection capacity of the 0.8 × 0.8 mm^2^ SoG electrode is estimated to be 5.2 μC/cm^2^, well within the capable range of a large-area electrode used in clinical stimulations.^61^

Figure 5c illustrates an *in vivo* nerve stimulation experiment in which surface ring electrodes were wrapped around the left leg to record the compound motor action potential (CMAP) from the gastrocnemius muscle in response to applied stimulation currents. The chemical inertness of SiC offers a wide electrochemical potential window of 2.8 V, indicating its suitability for electrophysiology recording and stimulation (Figure 5b). The CMAP amplitude shows the neurophysiological characteristics correlated to the number of activated motor axons from supramaximal electrical stimulation.^62^ To benchmark the nerve stimulation of the SiC electrodes, commercial bipolar gold electrodes (AD instruments) were used as the control (see Supporting Information, Figure S13). The stimulation for the SoG and the control electrodes was conducted under the same conditions of 1 ms-pulse-width at 1 Hz. Figure 5d shows the consistent evoked potential of the supramaximal signal at an input current of 250 μA. A CMAP response was first observed at 25 μA and 50 μA for SiC and control electrodes, respectively. This evoked CMAP response resulted from all of the activated motor axons—within the sciatic nerve—innervating the gastrocnemius muscle. When the stimulation current reached ∼250 μA (for either the SiC electrode or control), all motor axons were activated and the subsequent CMAP amplitude reached its supramaximal threshold. Accordingly, at a supramaximal stimulus, the CMAP amplitudes were 0.77 mV and 0.75 mV for SiC and control electrodes, respectively, in which the CMAP amplitudes saturated even when the stimulation current further increased (Figure 5e). The consistency of the recorded CMAP using our SoG electrodes versus the standard counterpart along with its physical stability suggests the capability of multifunctional endoscopy-based therapies incorporating tissue ablation and neuron modulation. The experimental studies demonstrated the excellent transparency, biocompatibility, stability, and multifunctionality (i.e., therapeutic, stimulation, and visual observation) of the present SoG bioelectronics compared with other bioelectronic material systems (Supporting Information, Figure S14). The proposed SoG platform has high potential for future translational research and scalable manufacturing due to its compatibility and integration with CMOS technology.

## Conclusion

This work presents a transparent, integrated, and multifunctional SiC-based bioelectronic system incorporating multiple biological sensors for tissue ablation applications. The excellent transparency of the SiC bioelectronic system, attached on the tip of surgical endoscopes, allows for direct observation and biological sensing of tissues during the ablation process. We presented proof-of-concept demonstrations using *in vitro* models for radiofrequency thermal ablation (with thermal generation) and irreversible electroporation (without thermal generation), with promising results for minimally invasive tissue ablation. The experimental results were consistent with finite element analysis models of the electric field distribution and bioheat equation. The biocompatibility and *in vitro* cell lysis using the SoG bioelectronics were also demonstrated in which cell lysis occurred at relatively low input power. The nerve stimulation from an *in vivo* experiment on an animal model demonstrated the capability of SiC electrodes for evoking muscle action potentials by modulating input current. Our findings indicate the potential development of robust, transparent, multifunctional, and scalable bioelectronic systems utilizing the excellent biocompatibility, robustness, and electronic/micromachining functionalities of the presented SoG platform.

## Methods

### Fabrication of SiC Bioelectronics

Conventional MEMS microfabrication steps were employed (i.e., metal sputtering, photolithography, spin coating, inductive coupled plasma (ICP) etching) to realize the SiC electrodes and sensor elements. Step-by-step fabrication processes was detailed in the Supporting Information, section 2.

### Optical Transparency Measurement

Laser sources with a wavelength from 400 to 800 nm were sourced from a Nanospec AFT 210 system passing through the SoG devices. Subsequently, relative recorded light intensity, with respect to direct light intensity, was used to calculate the optical transmittance in percentages.

### Temperature Sensor Calibration

The experiment was conducted in an enclosed temperature-controlled chamber. The electrical connections were wire bonded to a printed circuit board (PCB) and then connected to a Keithley 2450 SourceMeter to record resistance change upon varying the temperature from 25 to 80 °C. A schematic illustration can be found in Supporting Information, Figure S8.

### Impedance Measurement

A phantom tissue was prepared for impedance measurements. The details of the hydrogel preparation are described in the Supporting Information, section 4. A pair of electrodes were connected to a programmable LCR meter (i.e., HP 4284A). For impedance characteristics, an AC frequency range of 100 Hz to 1 MHz was scanned to obtain associated impedance values when the electrodes were in conformal contact with the phantom tissue. In contact and mapping measurements on the phantom tissue model, an AC frequency of 10 kHz was used. The impedance significantly increases when the electrodes form conformal contacts with the tissue model (i.e., 4 orders of magnitude higher than in the noncontact mode). For the viability measurement, an AC frequency of 10 kHz was also employed to measure the impedances with the initial hydrogel and the dried ones (heated at 50 °C for 30 s). A significant increase in the impedance was measured when the hydrogel dried out after 30 s.

### Thermal Imaging

An infrared (IR) thermal camera (Xenic Obi 384) with a spatial solution of 25 μm was placed on top of the SiC chip while in conformal contact with the phantom tissue model. The phantom tissue was cut into thin pieces to ensure good thermal observation from the camera. The thermal images and transient temperatures during the RFA process were recorded with associated software (i.e., Xeneth64).

### Radiofrequency Ablation

A sinusoidal AC at a frequency of 450 kHz, from a function generator (i.e., Agilent 33210A), was applied to a pair of electrodes. The applied AC voltage range is chosen from 10 V (min) to 40 V (max) corresponding to applied power from 0.15 to 2.4 W. The transient temperature during the RFA was recorded with the thermal camera and its associated software. For all applied voltages, the targeted temperatures were sufficiently achieved only after about 10 s. The voltage of 40 V yields a maximum temperature of approximately 80 °C. For demonstration, an AC voltage of 40 V was used to thermally ablate chicken breast lesions, which changed its color, which can be observed by the naked eye.

### Irreversible Electroporation

A biphasic voltage at a frequency of 1 Hz and pulse length of 100 μs was applied to a pair of electrodes. The voltages were generated from the function generator and then amplified using FLC F20A. When the electrodes are in conformal contact with the phantom tissue, to achieve sufficient electric field strengths in the targeted site, voltages with peak-to-peak values from 50 to 200 V were used. The thermal imaging setup was also used to observe the temperature variation. Yellow potatoes were used for the IRE demonstration: the ablated lesions were clearly observed with grooves formed in the sites contacting the electrodes.

### Cell Culture and Lysis

The eluate method was used to seed the cells in the mutilwell plates until confluence. Subsequently, the growth medium was substituted with a control medium (i.e., supernatants) with or without 0.1% H_2_O_2_ acting as the control while cells were incubated for 24 h, 48 h, and 5 days. At each time point, the cells were fixed with 4% formaldehyde then stained with ActinGreen 488 and NucBlue ReadyProbe reagents (Thermo Fisher Scientific) and then imaged using a fluorescent microscope (Nikon Eclipse Ti2). To assess the cell proliferation, cell numbers were counted with a colorimetric method using a cell counting kit (Abcam WST-8) dye mixed and kept incubated at 37 °C.

### Sciatic Nerve Stimulation

All of the procedures were approved by the Griffith University’s Animal Research Ethics Committee (Protocol number: ENG/01/21/AEC). Surgery was performed on the left sciatic nerve. The rats were anaesthetized with an O_2_/isoflurane mixture (30% /1–3%). Rats were placed prone over a heat blanket, and limb stabilization was achieved via an adhesive tape. The surgical site was shaved away at the left gluteal region. In addition, fur covering the left leg was removed with a commercially available hair-removal cream to achieve greater surface contact with the recording electrodes. The sensing surface ring electrode (black) was positioned where the gastrocnemius muscle has its maximum diameter. The reference surface ring electrode (red) is placed just beneath the sensing electrode. Contact gel was employed to optimize conductivity/transfer resistance. A skin incision was performed extending from a midpoint (between the hip joint and ischial tuberosity) to the knee. Blunt dissection was carried out (muscle splitting approach) using Iris scissors between the gluteus maximus and biceps femoris muscle. The sciatic nerve was identified under the gluteus maximus muscles. The nerve was isolated from the surrounding connective tissues and fascia using microscissors. The epineurium and its blood vessels were preserved. The SiC electrode was wrapped around the circumference of the sciatic nerve (Figure 5a). The nerve was electrically stimulated using rectangular pulses (duration 1.0 ms, repetition rate 1 Hz). Stimulation was carried out using a stimulus isolator (AD instruments FE180). Recordings were made and analyzed using a PoweweLab 4/26 channel recorder and LabChart software (ADInstruments).

## Supporting Information Available

Electrical stability in accelerated hydrolysis test, Fabrication process of SiC bioelectronics, Temperature sensing calibration, Phantom tissue preparation, Radio frequency thermal ablation modeling, Real-time temperature monitoring during tissue thermal ablation, Irreversible electroporation modeling, Biocompatibility of SoG, Cell lysis and electro impedance spectroscopy of SoG, Charge injection capacity measurement, Comparison of SiC and gold electrodes for nerve stimulation, Comparison of material platforms for bioelectronics (PDF)
