# Supplementary material for: Integrated, Transparent Silicon Carbide Electronics and Sensors for Radio Frequency Biomedical Therapy
Source: ACS Nano. 2022 Jul 11;16(7):10890–903. doi: 10.1021/acsnano.2c03188 (PMC9332346; doi:10.1021/acsnano.2c03188)
Supplement: Supplementary file 1 — nn2c03188_si_001.pdf [file nn2c03188_si_001.pdf]

Supporting information

Integrated, transparent silicon carbide electronics and sensors for radio-frequency biomedical therapy

Tuan-Khoa Nguyen,[1*] Sharda Yadav,[1] Thanh-An Truong,[1] Mengdi Han,[2] Matthew Barton,[3,4] Michael Leitch,[3] Pablo Guzman Duran,[1] Toan Dinh,[5] Aditya Ashok,[6] Hieu Vu,[7] Van Dau,[7] Daniel Haasmann,[1] Lin Chen,[8] Yoonseok Park,[9,10] Thanh-Nho Do,[11] Yusuke Yamauchi,[6,12] John A. Rogers,[9,13,*] Nam-Trung Nguyen,[1] Hoang-Phuong Phan[1,14*]

[1] Queensland Micro and Nanotechnology Centre, Griffith University, Queensland 4111, Australia

[2] Department of Biomedical Engineering, College of Future Technology, Peking University, Beijing, 100871, China

[3] School of Nursing and Midwifery, Griffith University, Queensland 4111, Australia

[4] Menzies Health Institute Queensland, Queensland 4222, Australia

[5] Centre for Future Materials, University of Southern Queensland, Queensland 4305, Australia

[6] Australian Institute for Bioengineering and Nanotechnology, The University of Queensland, Queensland 4072, Australia

[7] School of Engineering and Built Environment, Griffith University, Queensland 4215, Australia

[8] State Key Laboratory for Mechanical Behavior of Materials, School of Materials Science and Engineering, Xi'an Jiaotong University, Xi'an 710049, Shaanxi, People's Republic of China

[9] Querrey Simpson Institute for Bioelectronics, Northwestern University, Evanston, Illinois 60208, USA

[10] Department of Advanced Materials Engineering for Information and Electronics, Kyung Hee University, Yongin 17104, Republic of Korea

[11] Graduate School of Biomedical Engineering, The University of New South Wales, Sydney, New South Wales 2032, Australia

[12] JST-ERATO Yamauchi Materials Space-Tectonics Project, Kagami Memorial Research Institute for Science and Technology, Waseda University, Tokyo 169-0051, Japan

[13] Department of Materials Science and Engineering, Department of Mechanical Engineering, Department of Biomedical Engineering; Departments of Electrical and Computer Engineering and Chemistry, and Department of Neurological Surgery, Northwestern University, Evanston, Illinois 60208, USA.

[14] School of Mechanical and Manufacturing Engineering, The University of New South Wales, Sydney, NSW 2052, Australia

** Corresponding Author(s) k.nguyentuan@griffith.edu.au; jrogers@northwestern.edu; hp.phan@unsw.edu.au;*

KEYWORDS: Functional Endoscopy, Irreversible Electroporation, Radio Frequency Ablation, Thermal Therapy, Silicon Carbide, Bio-Integrated Electronics

1. Electrical stability in accelerated hydrolysis test

ITO thin-film resistors (sourced from Sigma Aldrich™, with the sheet resistance of 8-10 Ω/square) were prepared by sputtering Ti electrodes through a shadow mask and diced into $30\times5mm^2$ chips. Subsequently, they were soaked in 1X PBS solution at a temperature of 70 °C to validate their long-term stability and feasibility for high temperature RF ablation. Experimental results revealed that the resistance of ITO increased by approximately 250% after 5 days soaked in PBS at 70 °C, Figure S1(b). This significant change in electrical conductivity of ITO is attributed to a combination of the hydrolysis reaction, the absorption of water molecules, and the diffusion of ions from the biofluid into the film. This is in stark contrast to that of SiC in the soaking test at 96°C, Figure S1(b).

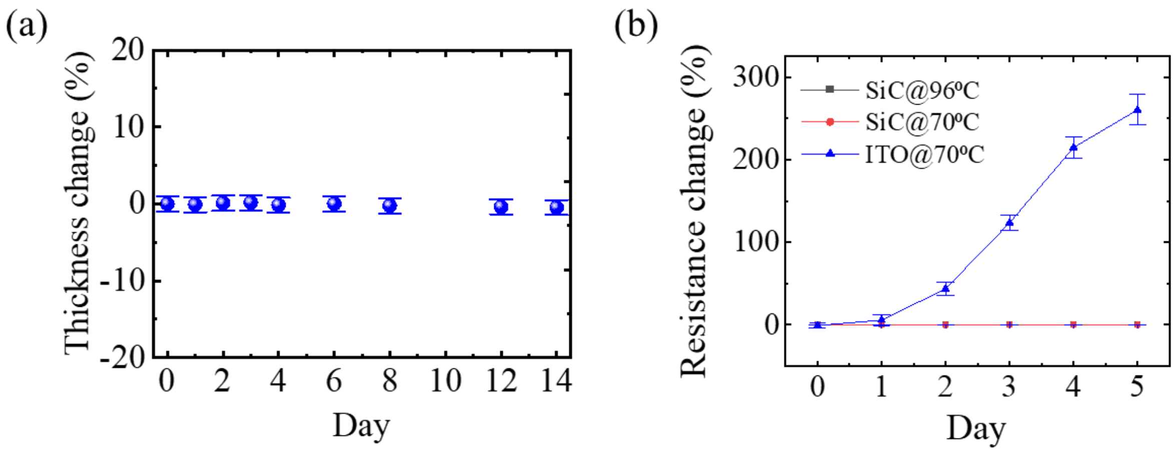

Figure S1. a) SiC thickness measurement in a hydrolysis test for up to 14 days at 96 °C. The recorded change is almost negligible. b) Comparison of resistance change in an accelerated soaking test in PBS 1X for up to 5 days at high temperature for SiC and ITO. The resistance of SiC remained almost unchanged whereas that of ITO varied dramatically up to 250%.

2. Fabrication process of SiC bioelectronics

The fabrication process for the SiC bioelectronics systems started with the growth process of SiC on a Si wafer with SiC and Si thicknesses of 600 nm and 500 µm, respectively. Figure S2 shows the TEM (a) and SAED images to verify that single crystalline 3C-SiC(100) has been grown. The AMF image indicated an excellent uniformity and smoothness of as grown SiC film on Si, Figure S3. The root-mean-square roughness of approximately 3nm is obtained, and the carrier concentration is measured to be in the range of 5×10^{19} cm^{-3}. The wafer was then bonded to a glass substrate followed by a polishing and chemical wet etching to remove Si carrier wafer.[1] To pattern SiC and contact paths on a new substrate (i.e., glass), first, 1.2 µm-thick positive photoresist was coated on the wafer using a spin coater at a speed of 4000 rpm. This photoresist was then soft baked at 105 °C in 1 min followed by lithography exposure (MLA 150) then patterned in photoresist developer. To prepare for the SiC process, the as-patterned photoresist is hard baked in 3 mins at 120 °C. Next, inductive coupled plasma (ICP) etching was employed to pattern the SiC electrodes and sensors. In the etching process, the chamber pressure was set at 2 mTorr with the plasma power of 120 W. As the average etching rate using HCl was approximately 100 nm/min, the etching process was performed until SiC pattern is completely etched (600 nm). 300 nm thick aluminum layer was deposited using a CVD sputtering machine and then electrodes were formed with photolithography and wet etching processes using Al etchant. Finally, 10 µm polyimide encapsulation was coated then pattern to open the exposed electrode areas while covering the contact path for the electrical isolation.

ACS Publications
Most Trusted. Most Cited. Most Read.

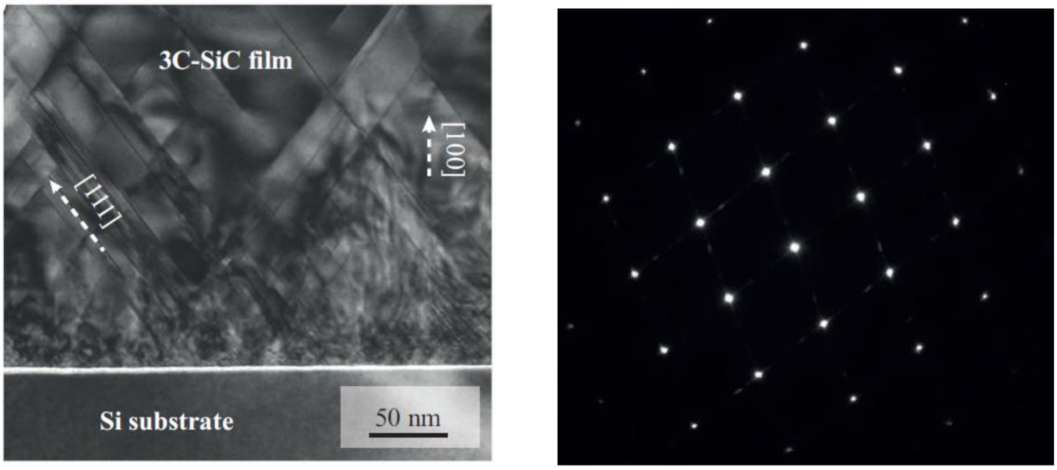

(a) (b)

Figure S2. TEM and SAED images for grown single crystalline 3C-SiC(100).

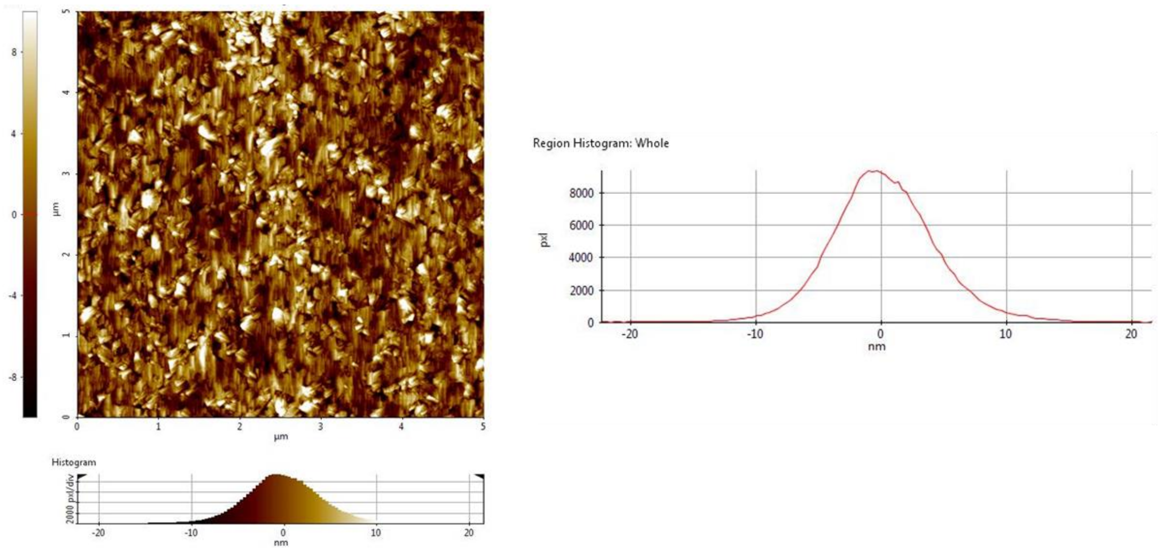

Statistics

Region	Min(nm)	Max(nm)	Mid(nm)	Mean(nm)	Rpv(nm)	Rq(nm)	Ra(nm)	Rz(nm)	Rsk	Rku
Whole	-22.303	21.527	-0.388	0.000	43.830	4.134	3.212	42.038	-0.188	3.734

Figure S3. Atomic force microscopy image of SiC material with the surface roughness of approximately 4 nm.

3. Temperature sensing calibration

To calibrate the integrated SiC temperature sensor, a enclosed temperature chamber was employed. The chamber temperature was varied from a test range from 20 °C to 80 °C monitored with a K-type thermometer (FLUKE^{TM} 714B) where K-type bead probe was placed closed to the SiC-on-glass chip, Figure S4.

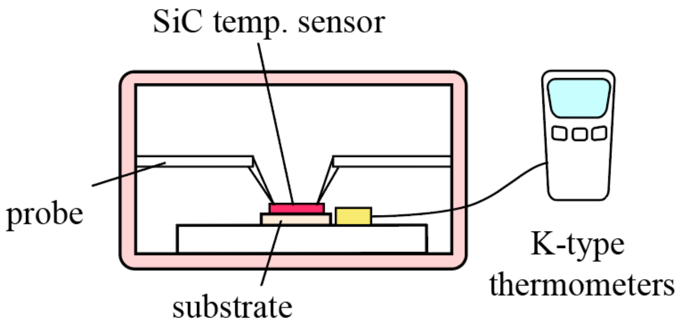

Figure S4. Temperature calibration for integrated temperature sensor using an enclosed chamber and K-type thermometer.

4. Phantom tissue preparation

Prepare the buffer and weigh the agarose gel powder (as per the requirement)

1. Mix agarose powder with PBS solution in a microwavable flask.
2. Dissolve the agar by heating the solution for intervals of 15-20 seconds in a microwave oven.
3. After each interval, remove the flask and gently swirl around to disperse the contents.
4. After 1-3 mins (with 15-20 s interval), the agar is completely dissolved in the solution. The heat interval was used to avoid overboiling the solution since the fast evaporation of buffer can lead to changes in the agar concentration in the gel.
5. Pour the solution to a petri dish and avoid causing bubbles that can disrupt the gel, then keep the mixed gel at room temperature for 1 hour, wait until completely solidified.
6. Cure the gel in air for 20 mins.

5. Radio frequency thermal ablation modeling

In the present study, RF thermal ablation was modelled by COMSOL™ Multiphysics, and the obtained results are shown in Figure S5. During the tissue ablation, the temperature distribution at different applied sinusoidal voltage at the middle point of upmost tissue surface presents similar trend, as shown in Figure S5(a). During the initial seconds, there exists a quick temperature rise, then followed by a steady platform. Simulation results showed that evaporation related dissipation had minor impact on tissue ablation. Approximately, scaling law for temperature rise dictates:

$$\frac{\kappa\left(T-T_0\right)}{\sigma V_0^{\ 2}}=f\left(\frac{S}{L},\frac{x}{L},\frac{y}{L},\frac{z}{L}\right) \qquad \text{(eq. 1)}$$

where κ, σ, S, L, V_0, and T_0 is thermal conductivity, electrical conductivity, electrode size, electrode spacing, applied potential, and initial temperature, respectively. The related scaling behavior of model parameters at the middle point of upmost tissue surface was shown in Figure S5(b) & (c). A perfect linear relationship between σV_0^2 and $\kappa(T - T_0)$ exists whenever we change σ from 0.05 to 0.3 S/m or V_0 from 5 to 40 V. In addition, the scaling behavior between S/L and $\kappa(T - T_0)/\sigma V_0^2$ was validated as parabolic function.

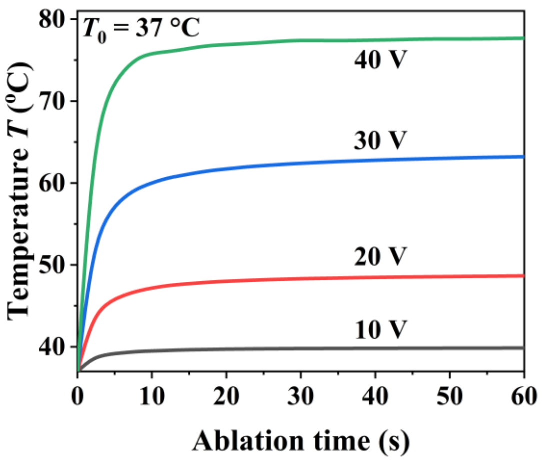

(a) temperature curve

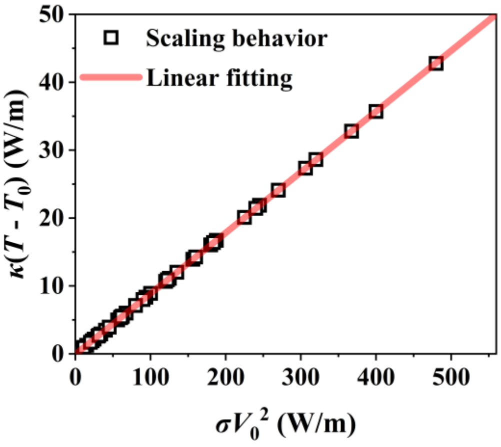

(b) scaling behavior of σV_0^2

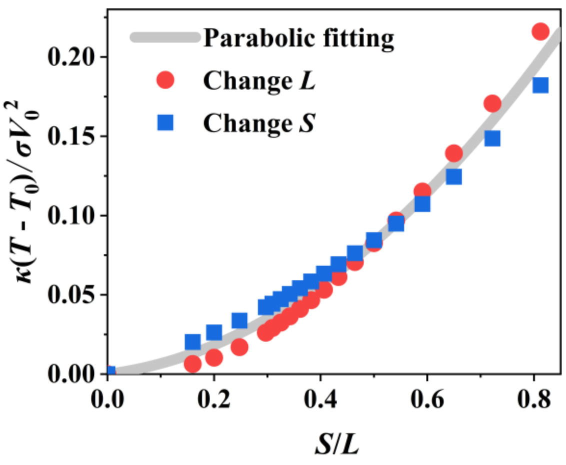

(c) scaling behavior of *S*/*L*

Figure S5. Temperature distribution at different applied sinusoidal voltage (a) and related scaling behavior of model parameters (b) and (c) at the middle point of upmost tissue surface.

6. Real-time temperature monitoring during tissue thermal ablation

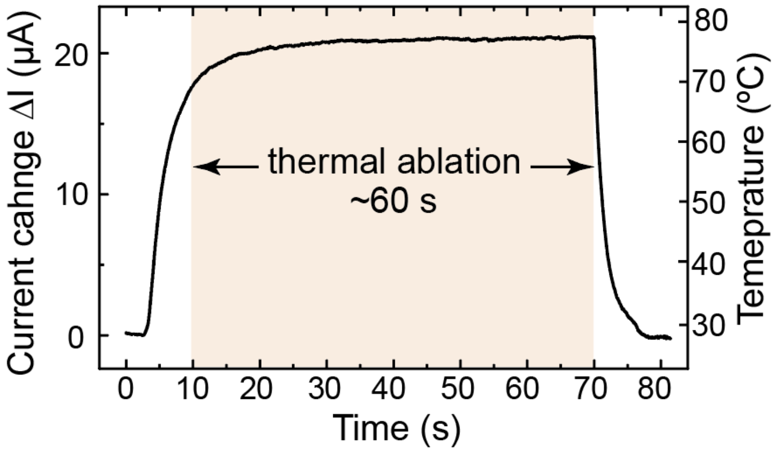

Figure S6. *In vitro* temperature monitoring during thermal ablation of a rat heart tissue for 60 s at the constant voltage mode to the sensor (V_{in}=1 V). The applied power to thermal ablation electrodes is approximately 2 W.

7. Irreversible electroporation modeling

The irreversible electroporation was also simulated with the electric field distribution shown in Figure S7. During the electroporation, the electric field was excited in dimensional analyses to obtain the scaling law for electric field strength as:

$$\frac{E}{V_0/L} = f\left(\frac{S}{L},\frac{x}{L},\frac{y}{L},\frac{z}{L}\right) \qquad \text{(eq. 2)}$$

Where S, L, and V_0 is electrode size, electrode spacing, and applied potential, respectively. The validation of the scaling law for the electric field strength was shown in Figure S8. As such, with constant S/L, the electric field strength changes linearly with V_0/L, Figure S8(a). Furthermore, the dimensionless electric field strength $E/(V_0/L)$ varies parabolically with S/L, Figure S8(b).

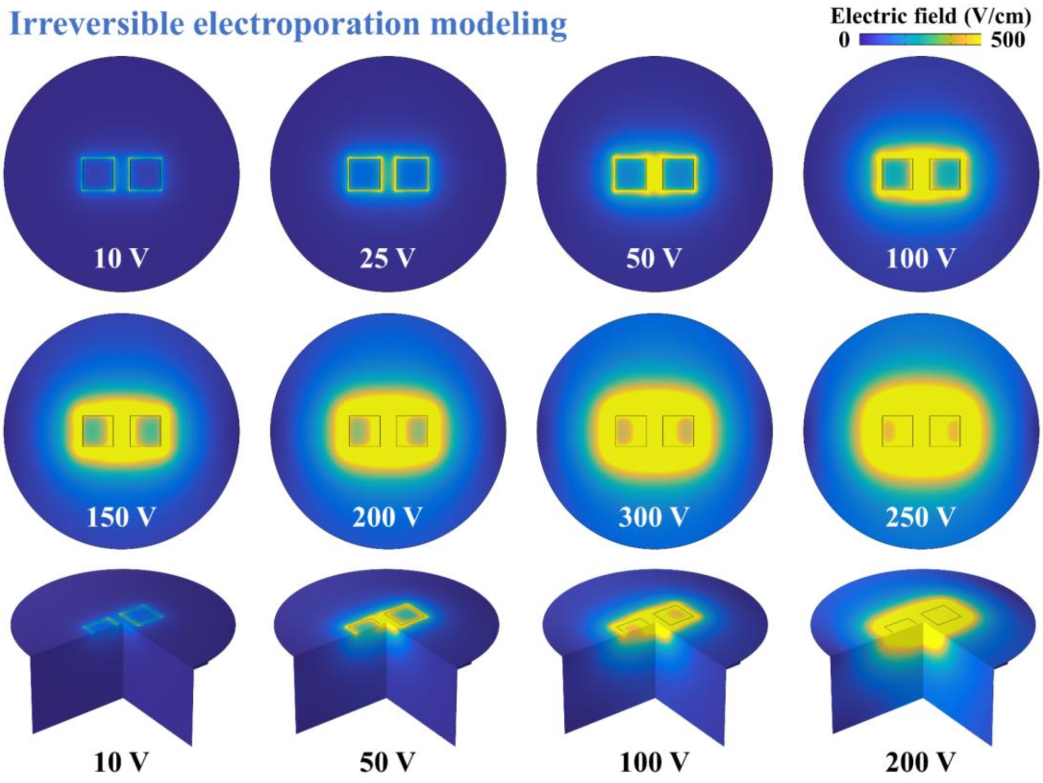

Figure S7. Electric field distribution in IRE at applied voltages of 10V, 50V, 100V, 200V.

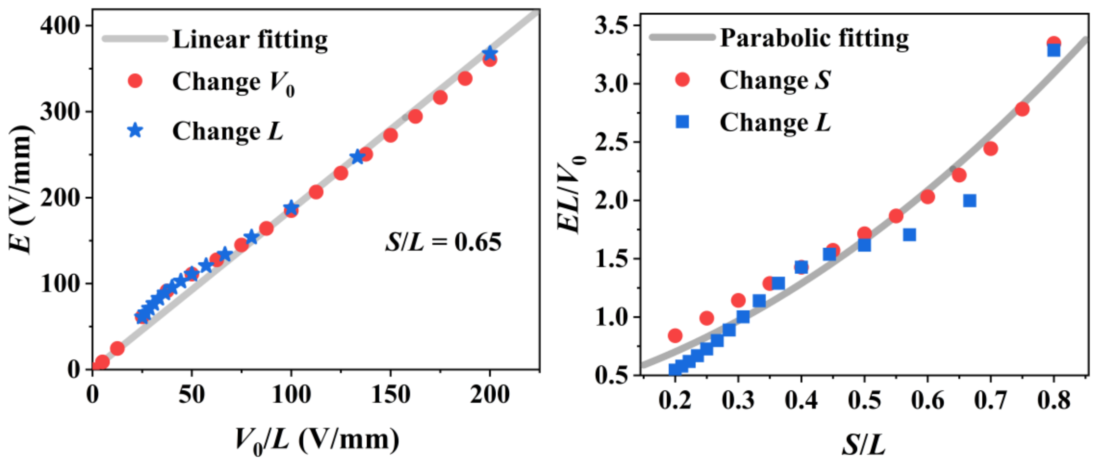

(a) scaling behavior of V_0/L (b) scaling behavior of S/L

Figure S8. Scaling behavior of electric field strength related to model parameters at the middle point of upmost tissue surface.

The irreversible damages typically occur when the electric field intensity exceeds E_c = 50 V/mm. Based on this, the scaling law for lesion size in potato model during the bipolar electroporation is given by:

$$\begin{cases} D/L = g_1\left(\frac{S}{L}, \frac{E_c L}{V_0}\right) \\ A/L^2 = g_2\left(\frac{S}{L}, \frac{E_c L}{V_0}\right) \\ V/L^3 = g_3\left(\frac{S}{L}, \frac{E_c L}{V_0}\right) \end{cases} \quad \text{(eq. 3)}$$

where D, A, and V represent damage length along the tissue middle line, area at the tissue upmost surface, and volume, respectively. The FEA validation of the scaling law shown in Figure S9 suggests good consistency despite changing geometry parameters can cause computation error (e.g., mesh difference among different cases).

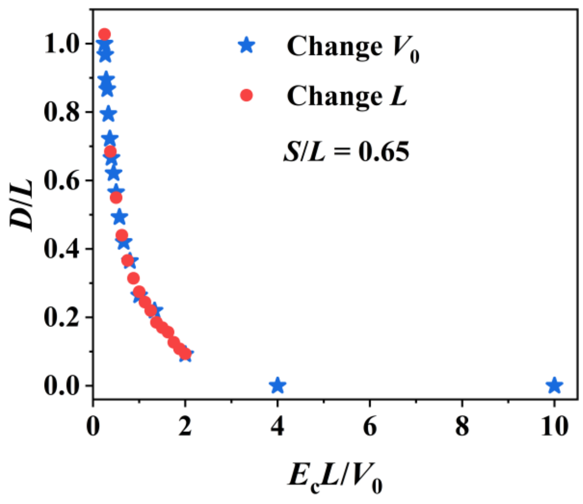

(a) D/L vs E_cL/V_0

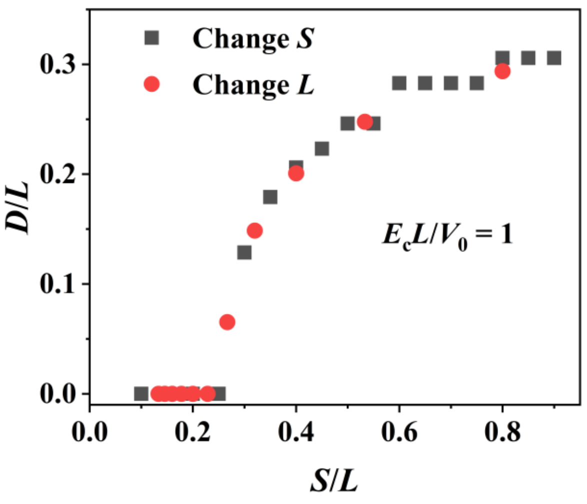

(b) D/L vs S/L

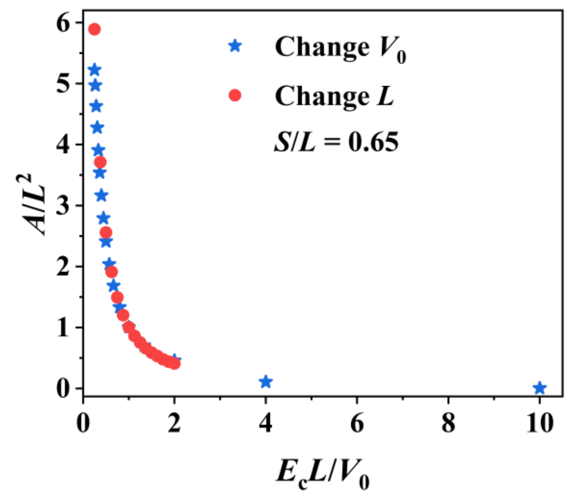

(c) A/L^2 vs E_cL/V_0

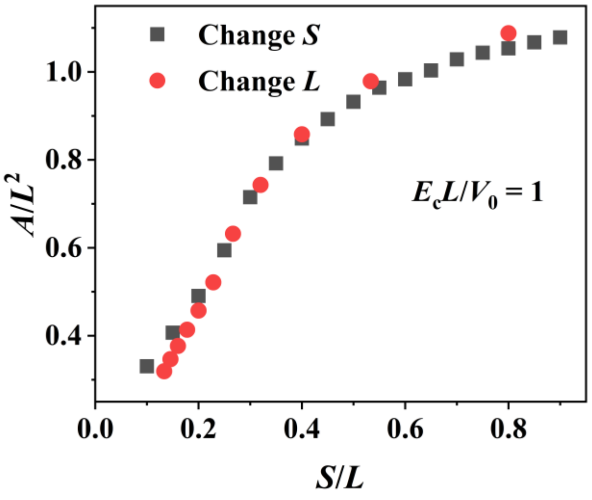

(d) A/L^2 vs S/L

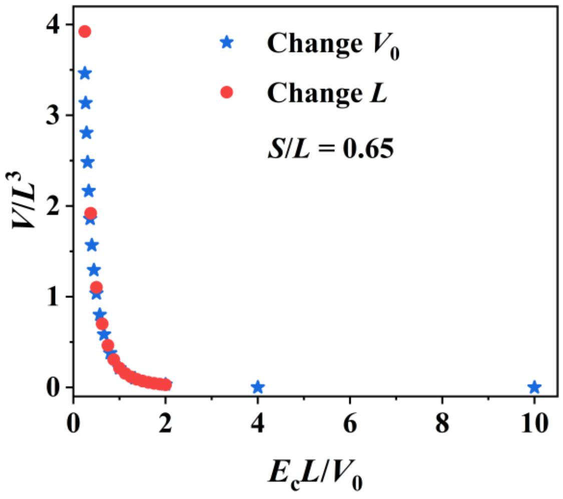

(e) V/L^3 vs E_cL/V_0

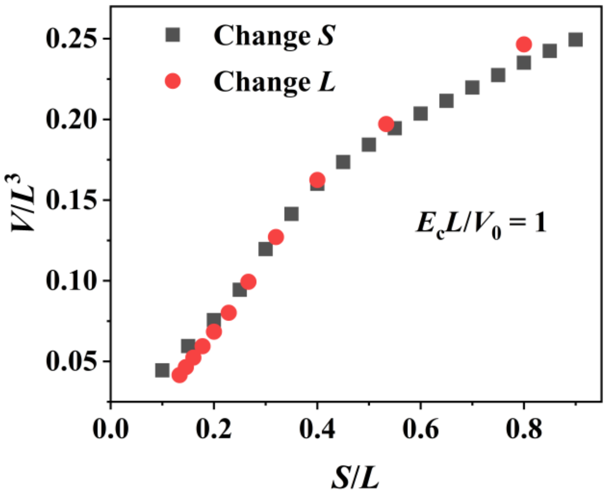

(f) V/L^3 vs S/L

Figure S9. Scaling behavior of irreversible damage related to model parameters.

8. Comparision of biocompatibility of SiC-on-glass versus standard cell culture substrate

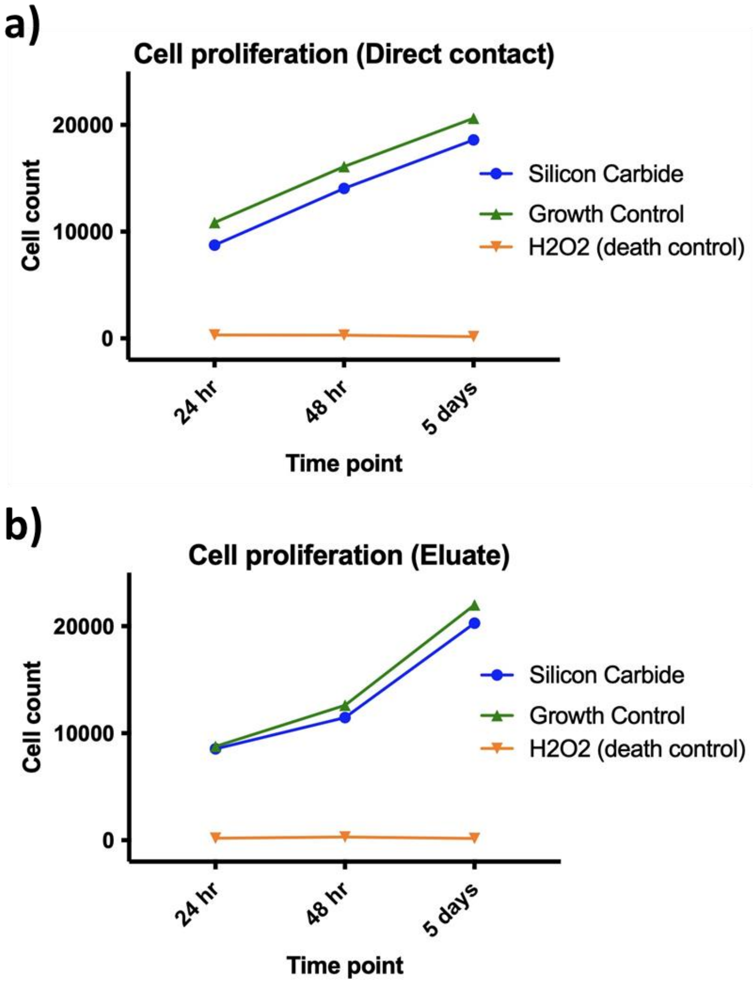

Figure S10. a) Comparison of cell proliferation on direct contact with silicon carbide, growth control and 0.01% H_2O_2 (death control). The cell count was done at indicated time points. b) Comparison of cell proliferation on eluate of silicon carbide, medium control and 0.01% H_2O_2. The cell count was conducted periodically at indicated time points.

9. Cell lysis and electro impedance spectroscopy of SiC-on-glass demonstration with direct obsevation

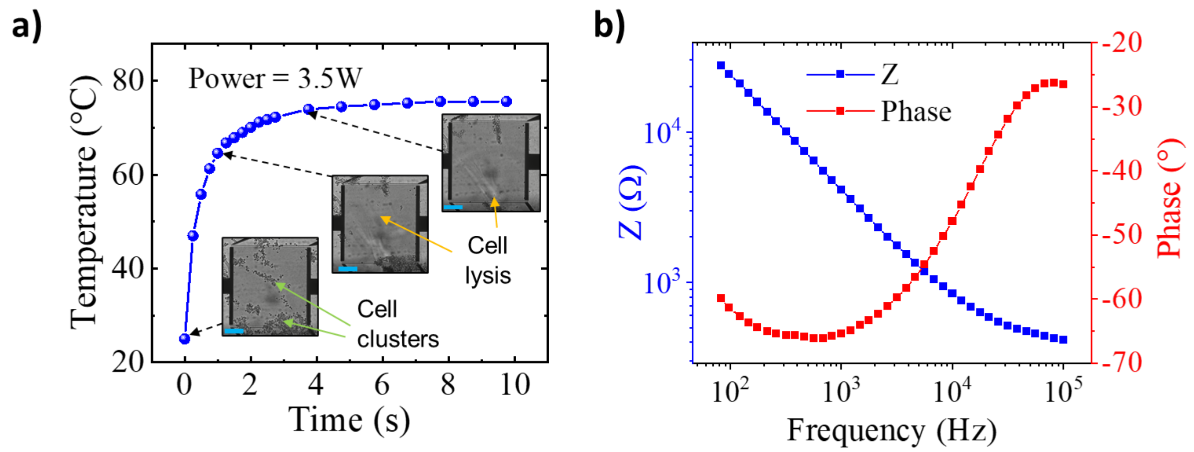

Figure S11. a) *In vitro* cell lysis: lysis project was visually observed by a fluorescent microscope after the 1s of applying voltage then completed after less than 4s. Inset: scale bar, 200 µm. b) Electro impedance spectroscopy (EIS) of single crystal SiC electrodes: impedance and phase angle versus frequency from 80 Hz to 100 kHz.

10. Charge injection capacity measurement

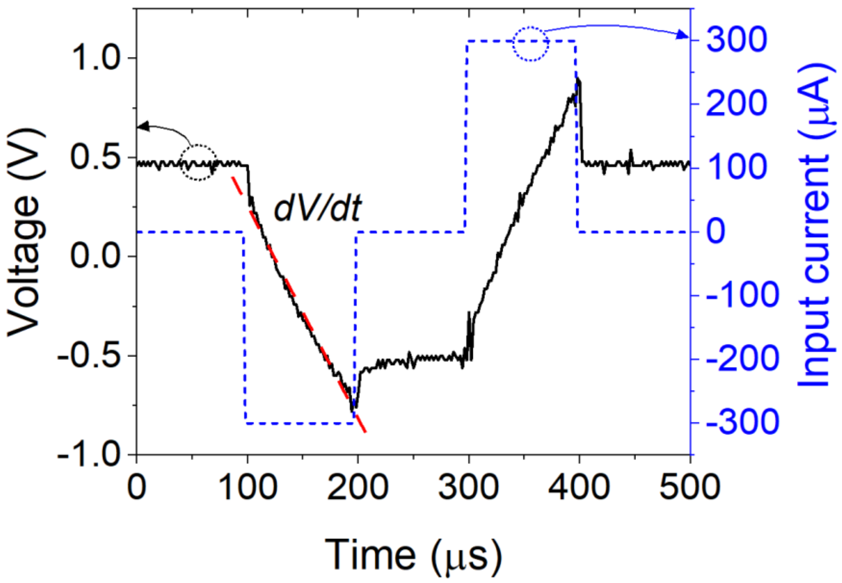

Figure S12. Transient voltage corresponding to an applied biphasic current with amplitude of 300 μA, 100 μs pulse width; the linear aggression (dV/dt, red dotted fit line) represents the electrode polarization during charge injection process, electrode size of 0.8×0.8 mm^2.

11. Comparision of SiC and control (gold) electrodes for nerve stimualtion

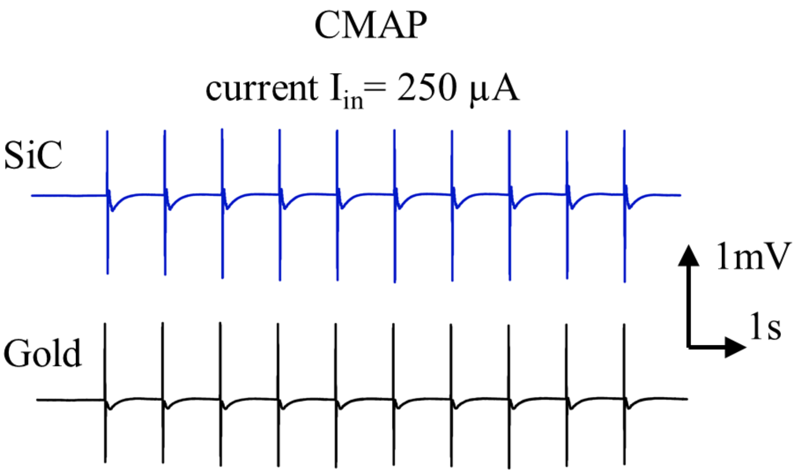

Figure S13. Comparison of recorded CMAP amplitudes at applied stimulating currents of 250 μA. SiC stimulation electrode demonstrates a similar performance compared with a standard gold electrode.

12. Comparison of material platforms for bioelectronics

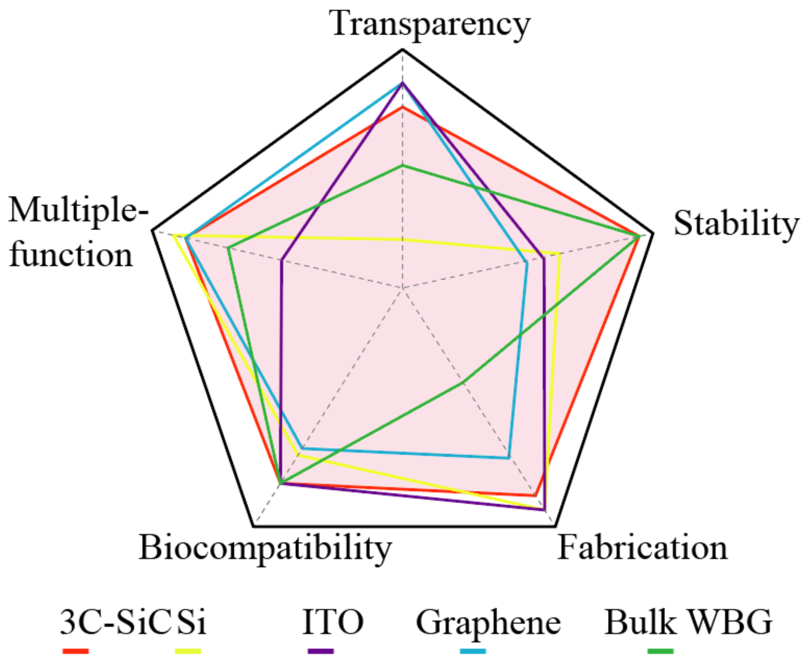

Figure S14. Comparison of the main characteristics of common bioelectronic systems for biological applications. WBG: wide band gap.

ITO is a degenerated n-type semiconductors, that exhibits good electrical conductivity, suitable for bioelectronics applications.[2] However, the high electrical conductivity also makes ITO behaving as metallic materials, which is nontrivial to modulate its physical properties through external stimuli such as photoenergy or strain engineering. To make ITO functioning as a semiconducting material, its thickness has to be reduced to an order of 10 nm, which correlates to the maximum depletion width (tens of nm for ITO field effect transistor channels).[3] However, as shown in Figure S1(b), the electrical resistance of ITO is relatively unstable in biofluid environment; therefore thinning down the film thickness will significantly reduce the device lifetime and stability. This drawback hinders the multimodalities of ITO for bioapplications. Si is the mainstream semiconducting materials with well-established fabrication technologies. However, as Si is not a transparent material, it is not suitable for biomedical devices where real-time observation is required. Two-dimensional materials have attracted significant research interest for a wide range of applications. Their excellent optical transparency places them as a

good candidate for bioelectronics electrodes and sensing components that allow optical observation. However, two dimensional materials typically exhibit highly active surface states, posing long-term stability issues when working under biofluid. In contrast, nanothin film SiC on glass can be engineered with an optimal conductivity such as highly doped for bioelectrodes (high current carrying capacity) and low doped for sensing elements (for sensitivity enhancements), along with excellent chemical stability. The fabrication processes for SiC nanothin films are highly compatible with conventional MEMS processes (i.e., dry etching of SiC), making it much more versatile compared to other SiC polytypes (e.g., 4H-, 6H-SiC) or bulk diamond like carbon. Micromachining for these polytypes is typically difficult due to the requirement of etching relatively thick SiC films (e.g., several hundreds of micrometer-thick). As an example, to form a 4H-SiC diaphragm, a top-down laser ablation method was introduced.[4, 5] However, the surface roughness of 4H-SiC was significantly higher (above 10 µm). Figure S14 highlights the advantages of our SiC-on-glass compared to other transparent counterparts in fabrication, integration, and functionality.

References

1. Phan, H. P.; Cheng, H. H.; Dinh, T.; Wood, B.; Nguyen, T. K.; Mu, F.; Kamble, H.; Vadivelu, R.; Walker, G.; Hold, L.; Iacopi, A.; Haylock, B.; Dao, D. V.; Lobino, M.; Suga, T.; Nguyen, N. T., Single-Crystalline 3C-SiC anodically Bonded onto Glass: An Excellent Platform for High-Temperature Electronics and Bioapplications. *ACS Applied Materials & Interfaces* **2017,** *9* (33), 27365-27371.

2. Kumar, A.; Zhou, C., The race to replace tin-doped indium oxide: which material will win? *ACS Nano* **2010,** *4* (1), 11-4.

3. Sakata, T.; Nishitani, S.; Saito, A.; Fukasawa, Y., Solution-Gated Ultrathin Channel Indium Tin Oxide-Based Field-Effect Transistor Fabricated by a One-Step Procedure that Enables High-Performance Ion Sensing and Biosensing. *ACS Appl. Mater. Interfaces* **2021,** *13* (32), 38569-38578.

4. Ransom, E. H.; Dowling, K. M.; Rocca-Bejar, D.; Palko, J. W.; Senesky, D. G. In *High-throughput pulsed laser manufacturing etch process for complex and released structures from bulk 4H-SiC*, IEEE MEMS, Las Vegas, IEEE: Las Vegas, 2017.

5. Nguyen, T.-K.; Phan, H.-P.; Dinh, T.; Dowling, K. M.; Foisal, A. R. M.; Senesky, D. G.; Nguyen, N.-T.; Dao, D. V., Highly sensitive 4H-SiC pressure sensor at cryogenic and elevated temperatures. *Mater. Des.* **2018,** *156*, 441-445.
